# Decomposing the spatial and temporal effects of climate on bird populations in northern European mountains

**DOI:** 10.1111/gcb.16355

**Published:** 2022-08-14

**Authors:** Ute Bradter, Alison Johnston, Wesley M. Hochachka, Alaaeldin Soultan, Jon E. Brommer, Elie Gaget, John Atle Kålås, Aleksi Lehikoinen, Åke Lindström, Sirke Piirainen, Diego Pavón‐Jordán, Tomas Pärt, Ingar Jostein Øien, Brett K. Sandercock

**Affiliations:** ^1^ Department of Terrestrial Ecology Norwegian Institute for Nature Research Trondheim Norway; ^2^ Cornell Lab of Ornithology Cornell University Ithaca New York USA; ^3^ CREEM, School of Mathematics and Statistics University of St. Andrews St. Andrews UK; ^4^ Department of Ecology Swedish University of Agricultural Sciences Uppsala Sweden; ^5^ Department of Biology University of Turku Turku Finland; ^6^ International Institute for Applied Systems Analysis (IIASA) Laxenburg Austria; ^7^ Finnish Museum of Natural History Helsinki Finland; ^8^ Department of Biology, Biodiversity Unit Lund University Lund Sweden; ^9^ Arctic Centre, University of Lapland Rovaniemi Finland; ^10^ BirdLife Norway Trondheim Norway

**Keywords:** anticipatory forecasts, climate decomposition, dynamic forecasts, forecast horizon, space‐for‐time substitution, spatiotemporal forecasts, spatiotemporal pattern, species distribution models, static forecasts

## Abstract

The relationships between species abundance or occurrence versus spatial variation in climate are commonly used in species distribution models to forecast future distributions. Under “space‐for‐time substitution”, the effects of climate variation on species are assumed to be equivalent in both space and time. Two unresolved issues of space‐for‐time substitution are the time period for species' responses and also the relative contributions of rapid‐ versus slow reactions in shaping spatial and temporal responses to climate change. To test the assumption of equivalence, we used a new approach of *climate decomposition* to separate variation in temperature and precipitation in Fennoscandia into spatial, temporal, and spatiotemporal components over a 23‐year period (1996–2018). We compiled information on land cover, topography, and six components of climate for 1756 fixed route surveys, and we modeled annual counts of 39 bird species breeding in the mountains of Fennoscandia. Local abundance of breeding birds was associated with the spatial components of climate as expected, but the temporal and spatiotemporal climatic variation from the current and previous breeding seasons were also important. The directions of the effects of the three climate components differed within and among species, suggesting that species can respond both rapidly and slowly to climate variation and that the responses represent different ecological processes. Thus, the assumption of equivalent species' response to spatial and temporal variation in climate was seldom met in our study system. Consequently, for the majority of our species, space‐for‐time substitution may only be applicable once the slow species' responses to a changing climate have occurred, whereas forecasts for the near future need to accommodate the temporal components of climate variation. However, appropriate forecast horizons for space‐for‐time substitution are rarely considered and may be difficult to reliably identify. Accurately predicting change is challenging because multiple ecological processes affect species distributions at different temporal scales.

## Graphical abstract

We separated variation in temperature and precipitation into spatial, temporal, and spatiotemporal components, and we modeled annual counts of 39 bird species breeding in the mountains of Fennoscandia over a 23‐year period. The directions of the effects of the three climate components differed within and among species, suggesting that species can respond both rapidly and slowly to climate variation and that the responses represent different ecological processes. Implicit in forecasts of species' distributions based on space‐for‐time substitution is the assumption of equivalent species' response to spatial and temporal variation in climate, which was seldom met in our study system.

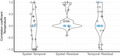

## 1 INTRODUCTION

Understanding the mechanisms and predicting the impacts of climate on the distributions and abundances of species is necessary for key goals in conservation and management. The complexity of species' responses to a changing climate may not be adequately characterized by the most commonly used form of forecasting based on species distribution models (SDMs, Adler et al., 2020; Illán et al., 2014; Rapacciuolo et al., 2012). In SDMs, associations are established between the occurrence or abundance of a species at sampling locations with climate and other environmental covariates (Franklin, 2009; Green et al., 2008; Jiguet et al., 2013; Stephens et al., 2016). Forecasts based on SDMs are typically implicitly based on *space‐for‐time substitutions*, which use current spatial patterns to forecast spatiotemporal patterns into the future (Adler et al., 2020; Blois et al., 2013; Stephens et al., 2016; Veloz et al., 2012). A spatial climate difference associated with variation in occurrence or abundance of a species is assumed to have the same effect as an equivalent change in climate through time at a single location. SDMs built with data from one time period and forecast or hindcast to a different period have given robust spatial predictions, but exceptions are common across a range of taxa, including birds (Araujo et al., 2005; Soultan et al., 2022), mammals (Davis et al., 2014), butterflies (Kharouba et al., 2009), and plants (Dobrowski et al., 2011; Pearman et al., 2008; Pearson et al., 2006; Veloz et al., 2012; Worth et al., 2014). Moreover, even for SDMs that accurately predicted future species distributions, occurrences or abundances at the sites where change occurred were often poorly predicted (Briscoe et al., 2021; Illán et al., 2014; Rapacciuolo et al., 2012). Systematic assessments are needed of the use of simple space‐for‐time substitutions versus more complex models of associations between species and environments.

**FIGURE 1:**
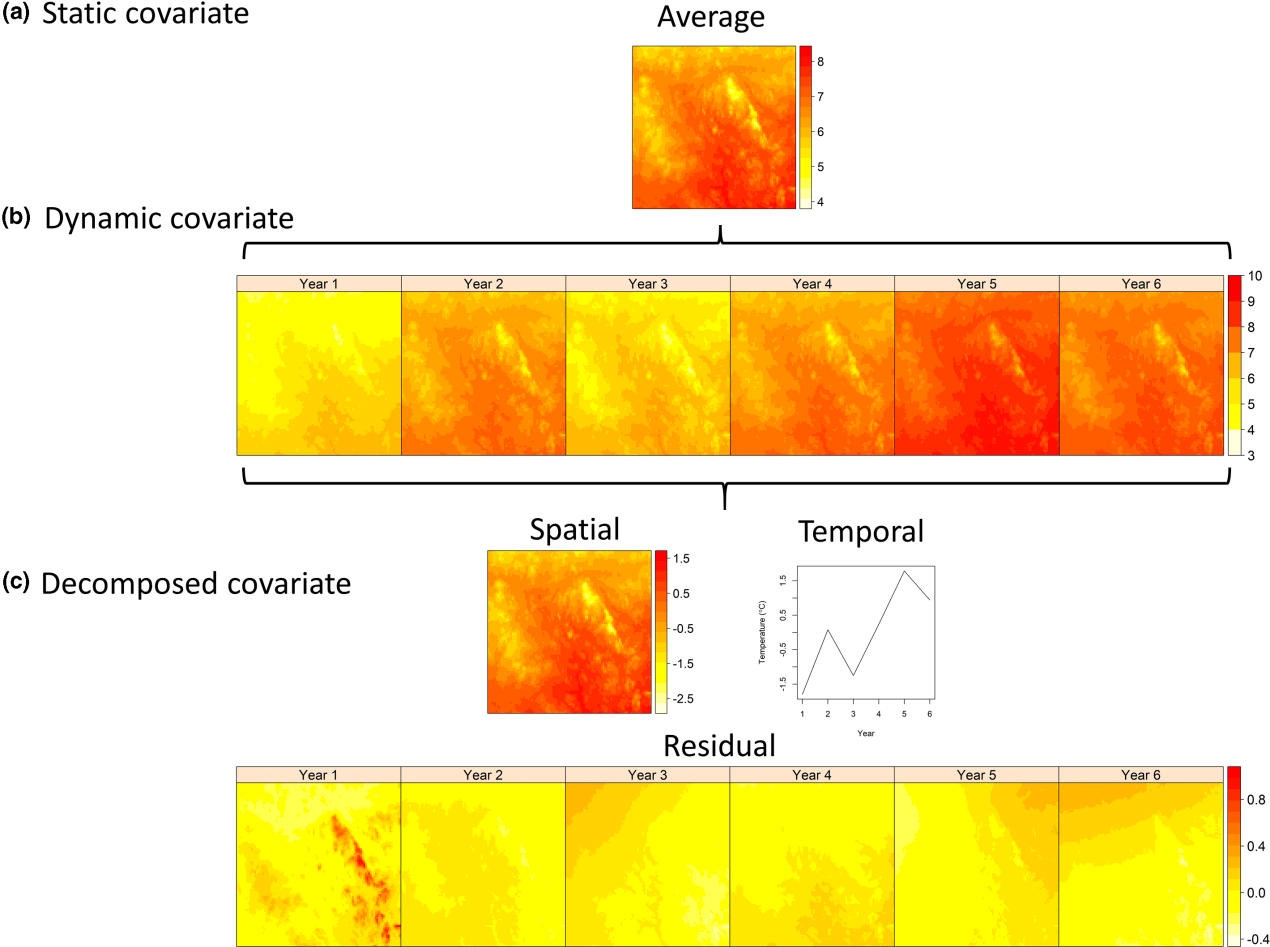
Static, dynamic, and decomposed covariates in species distribution models (SDMs). In SDMs with *static* covariates (a), time‐varying covariates such as temperature or precipitation are averaged over coarser temporal resolutions, such as several years. In SDMs with *dynamic covariates* (b), time‐varying covariates represent variation at finer temporal resolutions, such as per season or year, and are used to model corresponding seasonal or annual occurrence or abundance data. In SDMs with covariates *decomposed* into multiple components (c), time‐varying covariates are decomposed into the long‐term average spatial pattern, the temporal trend across the area of interest, and any residual (spatiotemporal variation). All three components are then used as covariates to model annual occurrence or abundance data.

**FIGURE 2:**
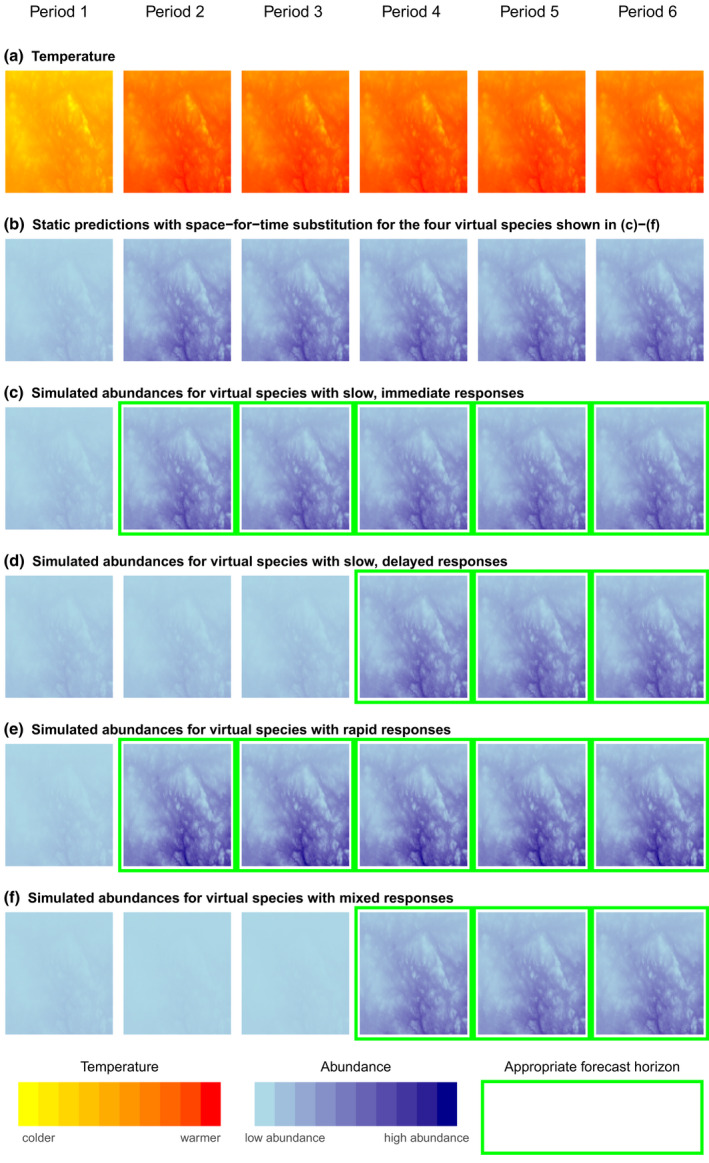
Conceptual figures illustrating the conditions under which static forecasts are appropriate when using space‐for‐time substitution. The average temperatures for several periods are shown (row a). Space‐for‐time substitution, based on identifying associations between species abundance and temperature in Period 1, will lead to identical predictions regardless of the rapidity of species' responses to annual variation in temperature in the future Periods 2–6 (row b). However, these predictions do not always match simulated species abundances, and green borders around panels in rows (c–f) show when space‐for‐time substitution produces valid predictions of species abundances. Applying static forecasts based on space‐for‐time substitution to species with immediate responses to changes in long‐term averages of climate (slow responses) is appropriate for as long as the species‐temperature relationship remains unchanged (row c). Applying static forecasts based on space‐for‐time substitution to species with slow, delayed responses (row d) is only appropriate after the delay has been overcome (from Period 4 onward) and for as long as the species‐temperature relationship remains unchanged. Forecasts are thus “left‐truncated” in time. Applying static forecasts based on space‐for‐time substitution to species with rapid responses (row e) is appropriate for as long as the species‐temperature relationship remains unchanged. In mixed responses found in this study and in Oedekoven et al. (2017), species respond both slowly and rapidly to temperature variation, but with the direction of effect for fast and slow responses being inconsistent. Applying static forecasts based on space‐for‐time substitution to species with mixed responses (row f) is only appropriate after the delay has been overcome (from Period 4 onward) and for as long as the species‐temperature relationship remains unchanged. Forecasts are thus “left‐truncated”. Changes in counts were based on simulated abundances (Methods S1). All forecasts are also “right‐truncated” (not shown), which means that at some point in the future, the current‐day relationship between species abundance and temperature will have changed due to an evolutionary response or another change. The time periods when the response lag is overcome or when the species‐temperature relationship changes are typically not known a priori.

An unresolved issue of *space‐for‐time substitution* is whether there may be a specific time period during which spatial and temporal species‐climate relationships are equivalent, which could explain some of the heterogeneity among species responses to climate variation. The time spans over which the climatic drivers of species distribution patterns are expected to act are reflected in the calculation of climate covariates for SDMs. Milanesi et al. (2020) distinguished between SDMs with *static* and *dynamic* covariates (Figure 1a,b). For the commonly used *static* covariates, covariates are averaged over several years or decades and the forecasted distribution is then an average distribution for a future time period (Araujo et al., 2005; Dobrowski et al., 2011). Longer forecast horizons have been advocated for two reasons: (1) the influence of stochasticity on shorter timescales, and (2) because changes in species' distributions may occur slowly (Blois et al., 2013; Pearman et al., 2008). Delayed changes in distributions may occur, for example, if dispersal limitations hamper the ability of a species to reach new suitable areas. Species' tolerances to climatic conditions may also be wider than the current realized niche, while responses to climatic change in the environment of the species may be slow, including succession from open habitats to forests or between forest types (Dobrowski et al., 2011; Schurr et al., 2012; Veloz et al., 2012; Wang et al., 2017; Zurell, 2017). If species' responses are delayed, valid forecast horizons based on space‐for‐time substitutions with static covariates will be left‐ and right truncated (Figure 2); forecasts would be applicable for the time period after the delay has been overcome (*left truncation*) and until the point in the future when the species‐climate relationship eventually changes (*right truncation*). Space‐for‐time substitutions based on static covariates have been suggested for forecasting changes occurring over longer time periods of decades up to millennia (Adler et al., 2020; Blois et al., 2013; Pearman et al., 2008).

**FIGURE 3:**
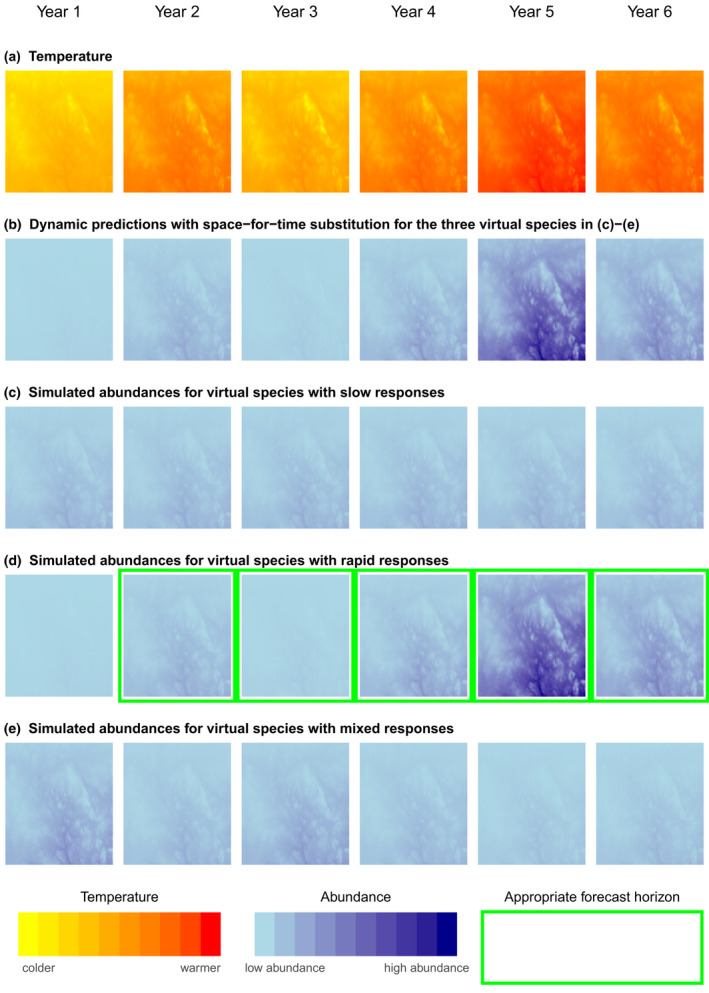
Conceptual figures illustrating the conditions under which dynamic forecasts are appropriate when using space‐for‐time substitution. Annual changes in temperature are shown (row a). Space‐for‐time substitution, based on identifying associations between species abundance and temperature, will lead to identical predictions regardless of the rapidity of species' responses to annual variation in temperature in the future Years 2–6 (row b). However, the predictions do not always match simulated species abundances, and green borders around panels in rows c–e show when space‐for‐time substitution produces valid predictions of species abundances. Applying dynamic forecasts based on space‐for‐time substitution to species with slow responses (row c) is inappropriate as forecasts based on the spatial pattern predict that species' abundances vary with temperature at fine temporal resolutions (row b), although variation in species' counts at fine temporal resolutions is stochastic. Applying dynamic forecasts based on space‐for‐time substitution to species with fast responses (row d) reproduces the year‐to‐year variation in the simulated abundances. In mixed responses, such as those found in this study and in Oedekoven et al. (2017), species respond both slowly and rapidly to different facets of temperature variation, but the direction of effects for fast and slow responses is inconsistent. Applying dynamic forecasts based on space‐for‐time substitution to species with mixed responses (row e) is inappropriate and does not correctly reproduce the year‐to‐year variation in the simulated abundances. All forecasts are also “right‐truncated” (not shown). Changes in counts were based on simulated abundances (Methods S1).

In contrast, Damgaard (2019) cautioned against forecasting based on space‐for‐time substitution unless species' responses to environmental changes occur relatively rapidly, because a changing environment may cause predictions to become unreliable. An example of a fast species response might be when local climate conditions determine the extent or water depth of wetlands and a wetland‐dependent species reacts quickly to the suitable habitat conditions. Here, we would expect the same relationship between the species' distribution or abundance and climate, regardless of whether the species‐climate relationship is in space or time or is describing the imminent or more distant future (Figures 2 and 3). Fast species responses can be modeled with *dynamic* covariates, in which covariates represent variation at fine temporal resolutions, such as seasons or years, and the resulting forecasts can incorporate rapid changes in distributions or abundances (Figures 2 and 3; Briscoe et al., 2021; Devenish et al., 2021). Thus, the underlying approaches of SDMs with static and dynamic covariates are different, with rapid effects of climate conditions omitted when using static covariates, but included via dynamic covariates. For rapid species responses and forecasts based on space‐for‐time substitutions, forecast horizons are right truncated, but not left truncated, because they encompass the entire time period from the immediate future until the species‐climate relationship changes (Figures 2 and 3). The time horizons apply to forecasts with either static or dynamic covariates (*static* or *dynamic forecasts*, henceforth).

The third type of SDMs, based on a *decomposition* of covariates (Figure 1c), provides a novel framework for investigation of the assumptions inherent in space‐for‐time substitution. Oedekoven et al. (2017) separated three components of climatic variation: (1) the long‐term average at each location (*spatial component*), (2) the annual variation across all locations (*temporal component*), and (3) all remaining variation (*residual component* or the spatiotemporal component or space‐time anomaly). Then, all components are included as covariates to model species counts or occurrences recorded at multiple sample sites and over multiple years. Oedekoven et al. (2017) found that the spatial effects of a given climate covariate were not always matched by equivalent effects of the temporal or residual components in modeling distributions of five common species of birds in Great Britain (ca. 210K km^2^, ca. 900 km latitude, 20 years).

**FIGURE 4:**
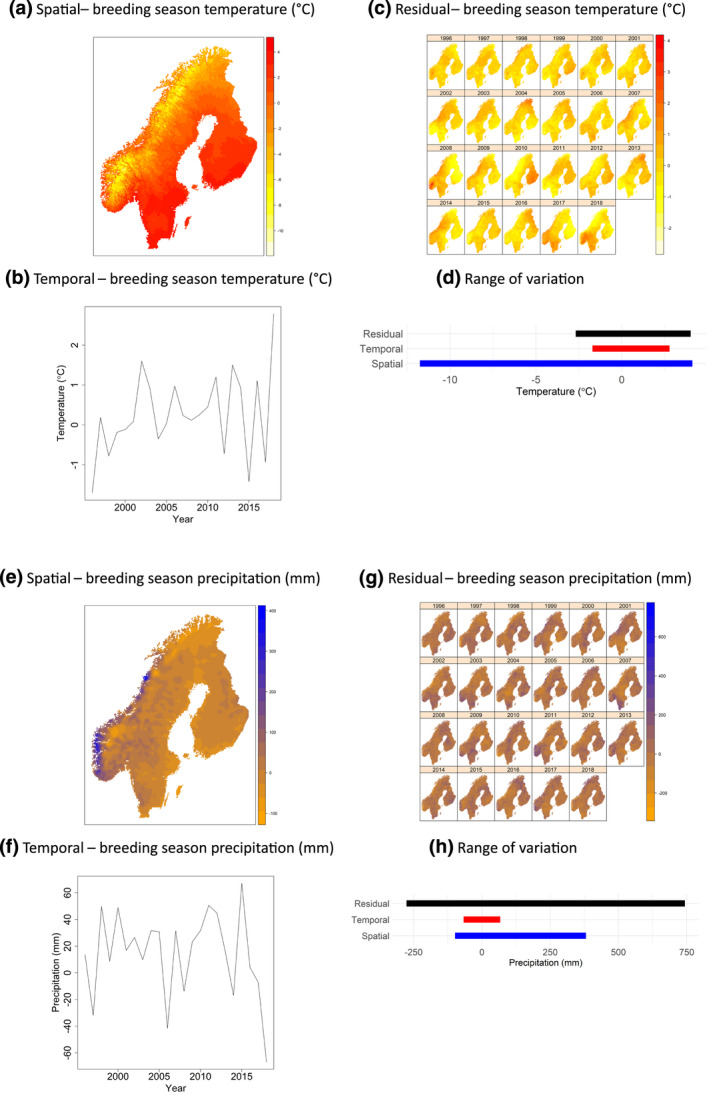
Spatiotemporal variation in temperature and precipitation in Fennoscandia, 1996–2018. The four panels on the top show the spatial (a), temporal (b), and residual (c) components of breeding season temperature (May–July) and (d) the range of variation per component. The four panels on the bottom show the spatial (e), temporal (f), and residual (g) components of breeding season precipitation (May–July), and (h) the range of variation per component. The plotted values show the difference in temperature and precipitation to the overall mean across all years and cells of the study area (10.7°C, 193 mm).

The aim of our study was to investigate equivalence in space and time of the relationships between local species abundances and climate for a diverse assemblage of bird species. A better understanding of whether and over which time periods the relationships between species abundance or occurrence and climate are equivalent in space and time is crucial because equivalence would provide greater confidence in forecasts. We applied SDMs with decomposed climate covariates to 39 species of birds breeding in the mountains of Fennoscandia (Figure 4) to obtain a better understanding of the spatial and temporal effects of climate. The study area is large with a complex biogeography comprising alpine, boreal, arctic, Atlantic, and continental regions (Roekaerts, 2002), including the highest mountains of Northern Europe and a large range of climate variability that is bounded by a temperate climate in the south, an arctic climate in the north, a maritime climate in the west, and a more continental climate in the east. Understanding the impact of climate variation on birds breeding in mountains and high latitudes is important given their vulnerability to climate warming (Freeman et al., 2018). We expected a similar response to climate across species for the spatial component because most bird species breeding in the mountains are expected to occupy the colder parts of Fennoscandia. Thus, the spatial component provides a useful baseline to compare relationships for the temporal and residual components. We considered the joint effects of temperature and precipitation on species distributions because both climatic variables can explain responses of mountain birds to a changing environment (Tingley et al., 2012). Specifically, for each species, we expected one of the following three outcomes, with consequences for time periods valid for space‐for‐time substitution:

1. Are the directions of effect between local abundance and climatic variation consistent in space and time? Equivalence of the direction of effect of the temporal and spatial relationship would support the assumptions underlying space‐for‐time substitution. Species' responses that lead to consistent directions of effects are rapid species responses to climatic variation. Additionally, consistent directions of effect could also be expected for slow species responses if the species is already responding to a change in the long‐term average climate. For rapid species responses, no left truncation of the forecast horizon is required for either static or dynamic forecasts (Figures 2 and 3). For slow species responses and static forecasts, left truncation of the forecast horizon is necessary, if the delay is not yet overcome. Dynamic forecasts based on space‐for‐time substitution are not valid for slow species responses, as short‐term variation is assumed to be stochastic (Figures 2 and 3).
2. Is local variation in species' abundances associated with temporal climate components? A lack of temporal effects would support the suggestion that species respond slowly to climate and have not yet responded to possible changes in the long‐term average climate. Consequently, left truncation may be necessary for static forecasts, while dynamic forecasts are not valid using space‐for‐time substitution (Figures 2 and 3).
3. Is local variation in species' abundances associated with temporal climate components, but the directions of effect between local abundance and climatic variation are not consistent in time and space? Species‐climate relationships that are inconsistent in time and space would indicate that species respond differently across different temporal scales of climate variation. Consequently, SDMs with static covariates do not comprehensively capture species‐climate associations. Static forecasts based on space‐for‐time substitution may need to be left truncated because an inconsistent pattern between space and time indicates that species responses to changes in the long‐term average climate will be delayed. Dynamic forecasts based on space‐for‐time substitution are not valid (Figures 2 and 3).

## 2 MATERIALS AND METHODS

### 2.1 Bird monitoring data

We used bird abundance data recorded on fixed routes of the three national bird monitoring schemes of Norway, Sweden, and Finland: the Norwegian terrestrial breeding bird monitoring (Norsk hekkefuglovervåking, tov‐e.nina.no/hekkefugl, 13 years, 2006–2018), the Swedish breeding bird survey (Svensk Fågeltaxering, www.fageltaxering.lu.se, 23 years, 1996–2018), and the Finnish breeding bird monitoring scheme (www.luomus.fi/en/bird‐monitoring, 13 years, 2006–2018). Fixed survey routes were laid out in a systematic grid across each country and are representative of the major habitats in Fennoscandia because they covered a large and topographically varied area across the three countries (ca. 1.1M km^2^, ca. 1800 km latitude, Figure 4). Routes were surveyed once each year between the end of May and early July, but not all routes were surveyed in all years. Survey routes were 6 km (Finland and Norway) or 8 km (Sweden) in total length, each typically a square or rectangle, and birds were recorded either along line transects (Finland) or on a combination of line transects and point count stations with 5 min survey durations (Sweden and Norway). Sections of a route were not surveyed if the terrain was inaccessible, but the total length of transects or the number of points surveyed was recorded to correct for occasional variation in sampling effort. In Sweden, birds were counted separately on point count stations and line transects. In Norway, all species were counted on point count stations, but a subset of rarer species were also counted on line transects. Consequently, we treated point counts and line transects as separate but spatially dependent surveys. The total numbers of individuals (Sweden) or pairs (Norway and Finland) detected were recorded per species for each route. Background details on each monitoring program are summarized by Kålås et al. (2014), Lehikoinen (2013), Lehikoinen et al. (2014), Lehikoinen and Virkkala (2016), and Ottvall et al. (2007) and in Methods S1. The Norwegian data were taken from 470 routes with both point count and line transect data, and an additional seven routes with only point count data (route‐years with transect data: 2765; route‐years with point counts: 3101). In Sweden, there were 716 routes with both transect and point count data (route‐years: 8897), and the Finnish data were from 563 routes with transect data (route‐years: 2845). In total, data from 1756 routes were included (routes with transect data: 1749; routes with point counts: 1186).

We initially selected 52 bird species for modeling, which were listed on the Swedish natural history portal Artfakta (artfakta.se) as occurring in the Fennoscandian mountains. We present results for a subset of 39 diverse species of birds (Table S1) for which our fitted models exceeded a performance threshold (see below): Anseriformes (four species), Galliformes (two species), Charadriiformes (17 species), Cuculiformes (1 species), and Passeriformes (15 species). The species ranged in body size from 8.7 g (willow warbler, *Phylloscopus trochilus*) to 1015 g (red‐breasted merganser, *Mergus serrator*) and contain invertivore, granivore, herbivore, omnivore, and aquatic predator species (Tobias et al., 2022). Most species are migrants and spend the summer breeding season in the Fennoscandian mountains (Svensson et al., 2009). Only five species are resident or only partially migratory: willow grouse (*Lagopus lagopus*), rock ptarmigan (*L. muta*), white‐throated dipper (*Cinclus cinclus*), northern raven (*Corvus corax*), and common redpoll (*Acanthis flammea*). Willow grouse and rock ptarmigan often move to more sheltered habitats and lower altitudes in winter. Common redpoll and white‐throated dipper are partial migrants that depart the most northerly areas of the study area in winter.

We jointly modeled data across all three countries, which ensured that sites spanned a larger range of environmental variation than in either of the three countries. Predicting outside of the sampled range of environmental variation can result in inaccurate predictions (Dormann et al., 2013; Randin et al., 2006). We conducted a validation analysis to confirm that our choice of spatial extent did not influence the modeled relationships with the spatial climate component (Figure S5). We modeled counts, whereas many previous studies have modeled species occurrence. Occurrence and abundance are positively associated, and abundance is a key quantity of interest for biodiversity studies (He & Gaston, 2003; Kunin, 1998). Abundance data contain more information than occurrence data (Johnston et al., 2015), and SDMs based on abundance data with predictions converted to occurrence can outperform SDMs based on occurrence data (Howard et al., 2014).

### 2.2 Environmental data

To model the influence of climate on the local abundance of birds during the breeding season, we used daily mean temperature and daily precipitation for the 48‐year period of 1971–2018 from the Nordic Gridded Climate Dataset (NGCD), provided by the Norwegian Meteorological Institute (Lussana, Saloranta, et al., 2018; Lussana, Tveito, et al., 2018). The NGCD is an interpolation of observed temperature and precipitation data for Norway, Sweden, and Finland onto a high‐resolution grid of 1 km. To control for the expected effects of habitat on bird abundances, we included land cover information from the European CORINE land cover data (European Union, 2019). CORINE data classify land cover into 44 classes at a 100 m spatial resolution. We pooled similar land cover classes into seven broader categories that were relevant for our focal species: (1) sparsely vegetated mountain areas (bare rocks, sparsely vegetated areas), (2) mountain vegetation (moors, heathlands, and natural grasslands), (3) deciduous forest, (4) other forest (coniferous and mixed forest and transitional woodland scrub), (5) wetlands (inland marshes and peat bogs), (6) inland waters, and (7) agriculture. CORINE land cover classifications were available at 6‐year intervals, for the years 2000, 2006, 2012, and 2018, and we matched bird counts from the periods 1996–2003, 2004–2009, 2010–2015, and 2016–2018 with the corresponding land cover data. We found relatively few changes between consecutive CORINE maps in mountain environments, and therefore, assigning bird counts to the nearest land cover map (≤4 years) should not introduce large errors. Last, elevation, slope, and solar radiation are important explanatory variables for distributions of many species (Franklin, 2009). We extracted elevation from the Copernicus digital elevation model (DEM, 25 m spatial resolution, European Union, 2019) and calculated slope and solar radiation from the DEM (Methods S1).

Species‐environment relationships are spatial scale dependent and covariates may influence a response variable at multiple spatial scales and potentially in different ways (Bradter et al., 2013; Wiens, 1989). We restricted our choice of spatial scale of covariates to one scale as our models already contained a large number of covariates due to the climate decomposition. We used 300 m buffers to represent the main activity areas used by the detected individuals during the breeding season. Thus, all environmental covariates were summarized within 300 m buffers around each point count station and either side of line transects. Separately across all point counts and along the full length of transects for each survey route, we calculated the proportion of each of the seven land cover categories, and the mean value for each climatic and topographical covariate.

To assess collinearity among the different covariates, we calculated variance inflation factors (VIFs, Zuur et al., 2009). Climate covariates and slope were included as covariates for each species. We removed elevation because the variable was correlated with temperature leading to a high VIF. Other covariates were selected based on existing knowledge of the habitat associations of each species and with the aim to keep VIFs between fitted covariates low (Methods S1). For each species, all VIFs between fitted covariates were <4.

#### 2.2.1 Climate decomposition

From the daily NGCD climate data, we calculated the mean temperature and cumulative precipitation during the breeding season within each 1 km^2^ for the years 1971–2018, inclusive. We investigated the effect of climate variation both of the current and the previous year because settlement decisions of individuals may be influenced by the current local conditions, but also by past conditions if individuals preferentially return to locations where they nested successfully in previous years (Schaub & von Hirschheydt, 2009; Shitikov et al., 2015). Additionally, environmental conditions in the previous year can influence recruitment into a population through the number of individuals available to settle (Pearce‐Higgins et al., 2015). For the current year, we investigated representing the breeding season as both May–July and May–June. The spatial component of May–July climate better represents the typical choice in SDMs with static covariates, where climate data are frequently aggregated over the breeding period. By contrast, the temporal and residual components of May–June better represent the temporal and spatiotemporal climate variation experienced by birds in a given year at the time when the surveys were conducted. We calculated both mean temperature and cumulative precipitation for each of these combinations of months and years.

Following Oedekoven et al. (2017), we decomposed the May–July and separately the May–June climate data into the spatial, temporal, and residual components. We conducted the decomposition in four steps. First, we calculated the *global mean* across the three countries and all years (1971–2018) for each climate variable (Temperature 10.7°C in May–July and 8.9°C in May–June; precipitation: 193 mm in May–July and 113 mm May–June) and centered the values of each climate variable across all years (1971–2018) and grid cells. Thus, for each climate variable *C* (breeding season temperature or precipitation), *i =* 1, …, *N* grid cells and *t =* 1, …, *T* years, we first calculated the global mean as:${\mathrm{CC}}_{i,t}=C_{i,t}-\frac{\sum_{t=1}^{T}\sum_{i=1}^{N}C_{i,t}}{N\times T}$

where *CC* is the centered climate variable and *C* is the uncentered climate variable. Next, the *spatial component* was calculated as the long‐term centered spatial average value for each of ca. 1.1 M 1‐km^2^ grid cells during the 48‐year period of 1971–2018 (Figure 4): $\text{CC}{\text{Space}}_{i}=\frac{\sum_{t=1}^{T}{\mathrm{CC}}_{i,t}}{T}$.

Then, the *temporal component* was calculated as the annual deviations during the 23‐year study period 1996–2018 from the long‐term average; deviations were calculated for each year by averaging centered values across all grid cells in all three countries (Figure 4): $\text{CC}{\text{Time}}_{t}=\frac{\sum_{i=1}^{N}{\mathrm{CC}}_{i,t}}{N}$.

Last, the *residual component* of climate variation was calculated as the spatiotemporal climate variation that remained after accounting for the spatial and temporal components. The residual component was calculated separately for each cell and year 1996–2018 (Figure 4): $\text{CC}{\text{Residual}}_{i,t}={\mathrm{CC}}_{i,t}-\text{CC}{\text{Space}}_{i}-\text{CC}{\text{Time}}_{t}$.

### 2.3 Statistical analysis

For each bird species, we fitted three different models, with each model including the three climate components from either: (1) May–July of the current year, (2) May–June of the current year, or (3) May–July of the previous year. Results were similar for the two models with climate components of the current year. Here, we present results with climate components from May–July in either the current or previous year.

We modeled counts of each species per survey route and per year (hereafter “local abundances”). Ecological count data are often overdispersed (Lindén & Mäntyniemi, 2011) and some focal species had many survey routes with zero counts. To identify the most appropriate way to account for potential overdispersion and zero‐inflation, we conducted preliminary analyses where we used a log link and four alternative candidate error structures for the models with climate components of the current year: a Poisson distribution, a negative binomial distribution (NB), and zero‐inflated versions of both models (ZIP and ZINB). The ZIP and ZINB are mixture models combining a Bernoulli distribution with either a Poisson or negative binomial distribution, respectively. Species‐specific model selection between these four distributions was based on rankings with AIC (Akaike's Information Criterion), and the model with the lowest AIC was chosen (Burnham & Anderson, 2004). For all bird species, the model with minimum AIC value was either the NB or the ZINB model. Due to problems with model convergence in some models for white‐throated dipper, we used a Poisson distribution for this species.

In addition to the habitat and topographical covariates (see above and Methods S1), we used the spatial, temporal, and residual components of both temperature and precipitation as covariates. We allowed for interactions between temperature and precipitation for each component of the climate decomposition. We accounted for temporal variation in abundances not explained by the covariates by fitting second‐order polynomials for year. We accounted for differences in protocols among the national monitoring schemes by including two categorical variables: (1) “Survey”, which could be either Point or Line to account for differences in abundances between point count and line transect methods; (2) “Unit”, with the levels Individual or Pair, to account for differences in counts due to recording observed birds as either individuals or pairs. We used the natural logarithm of survey effort (the length of the transect line or the number of point count stations per route) as an offset to account for differences in survey effort. To account for the repeated measures from the same routes in different years and between‐route differences not explained by the covariates, we fitted route as a random intercept. The same suite of covariates was fitted both to the count and the zero‐inflation part of the model, with the exception of the covariates for monitoring schemes which were not expected to influence the recording of zeros. We present results based on model selection using AIC (Methods S1). Results based on full models led to the same conclusions, and therefore, we do not present details related to model selection uncertainty. Despite the large extent of the study region and long‐term dataset, we had relatively few observations per year for some species (Table S1). Therefore, for rarer species, we expected the statistical power of our models to be lower and with higher uncertainty in model selection.

Our modeled abundances were relative indices rather than absolute abundances because we were unable to account for imperfect detection. The bird monitoring data did not contain the repeated counts necessary to estimate detection probability with occupancy models (MacKenzie et al., 2003), and only a subset of the sampling protocols used distance bands, which are necessary for distance sampling methods (Buckland et al., 2001). Nevertheless, survey methods were standardized, and counts were not carried out in rainy or windy conditions, and we assumed the ability of surveyors to detect birds was relatively constant among years. Potential differences in detectability by route were also accommodated by the random effect for route. Climate change has advanced the beginning of the breeding season of 73 species in Finland by an average of 4.6 days over four decades (Hällfors et al., 2020). By contrast, in Northern Sweden, only 3 out of 14 species advanced their breeding season over 32 years, though years with warmer temperatures in May led to earlier breeding of most species (Ram et al., 2019). Variability in phenology can impact species detectability, but effects in UK and Finnish monitoring schemes were relatively small (Lehikoinen, 2013; Massimino et al., 2021).

#### 2.3.1 Assessing the effects of the climate components

To assess the effects of spatial, temporal, and residual variation in climate on local relative abundance of a species, we predicted the relative abundance of each species at each of the 1749 routes of line transects while varying the values of temperature and precipitation for each component within their observed ranges in the study area (Figure 4d,h). We fixed all other covariates to typical values (Methods S1). To assess the effects of only the spatial climate components, we set the temporal and residual climate components to zero and created new combinations of values of covariates by varying temperature and precipitation for the spatial component along 10 evenly spaced values between the observed minimum and maximum values for the study region. Then, we made predictions from the model for all possible combinations of the 10 temperature and 10 precipitation values. To assess the effects of the temporal and residual climate components, we repeated the procedure, but holding the spatial component constant at observed values and varying the temporal or residual component. We then summed the route‐specific predicted abundances over all 1749 survey routes for each combination of temperature with precipitation and each climate component. Finally, we calculated Spearman's rank correlation coefficients (*r*_*s*_) between predicted relative abundances of: (1) the spatial and temporal components, (2) the spatial and residual components, and (3) the temporal and residual components. We calculated three sets of correlation coefficients: (1) along temperature and precipitation gradients, (2) along a temperature gradient, and (3) along a precipitation gradient (Figure S6).

#### 2.3.2 Model performance and robustness tests

To assess model performance, we computed the Pearson correlation coefficient between the fitted local abundances and the observed counts for each species across all routes, separately for Norway, Sweden, Finland, and then for all three countries combined. We present results for the 39 of 52 species of birds (75%) with at least an intermediate level of correlation in the models in which climate data were summarized for the current year: *r*_*p*_ ≥ 0.4 for each country and *r*_*p*_ ≥ 0.5 for Fennoscandia as a whole. The other 13 species were mainly rare species with many zero counts from the survey routes. We assessed how well the models generalized to predicting relative abundances by holding 1 year out during model fitting, and then by computing the Pearson correlation coefficient via a cross‐validation procedure (Wenger & Olden, 2012; Methods S1).

We assessed the sensitivity of our results to three elements of our data and models (Methods S1). First, we tested the robustness of our models to spatial and temporal sample selection bias. Our data were spatially and temporally biased because survey coverage in more remote areas tended to be sparser and monitoring started in different countries and regions in different years. Such biases can potentially bias the conclusions from models and forecasts (Bradter et al., 2018, 2021; Johnston et al., 2020). Second, we tested the robustness of our joint analysis of data from three national monitoring schemes. Last, we assessed robustness to residual spatial or temporal autocorrelation which can increase Type I error rates (Dormann et al., 2007). Our three sets of robustness tests indicated that conclusions from the models were robust to the spatial and temporal bias in the data and to the joint analysis of data from the three national monitoring schemes (Methods S1). Thus, we present findings based on data from the full time series and all three countries.

### 2.4 Software

All analyses were conducted in R 4.1.2 (R Core Team, 2021). VIFs were calculated using the function corvif from Zuur et al. (2009). Models were fitted using package glmmTMB (Brooks et al., 2017). Model validation was aided by functions from package DHARMa (Hartig, 2018). We assessed spatial autocorrelation of model residuals with function Moran.I from the package ape (Paradis & Schliep, 2019).

## 3 RESULTS

**FIGURE 5:**
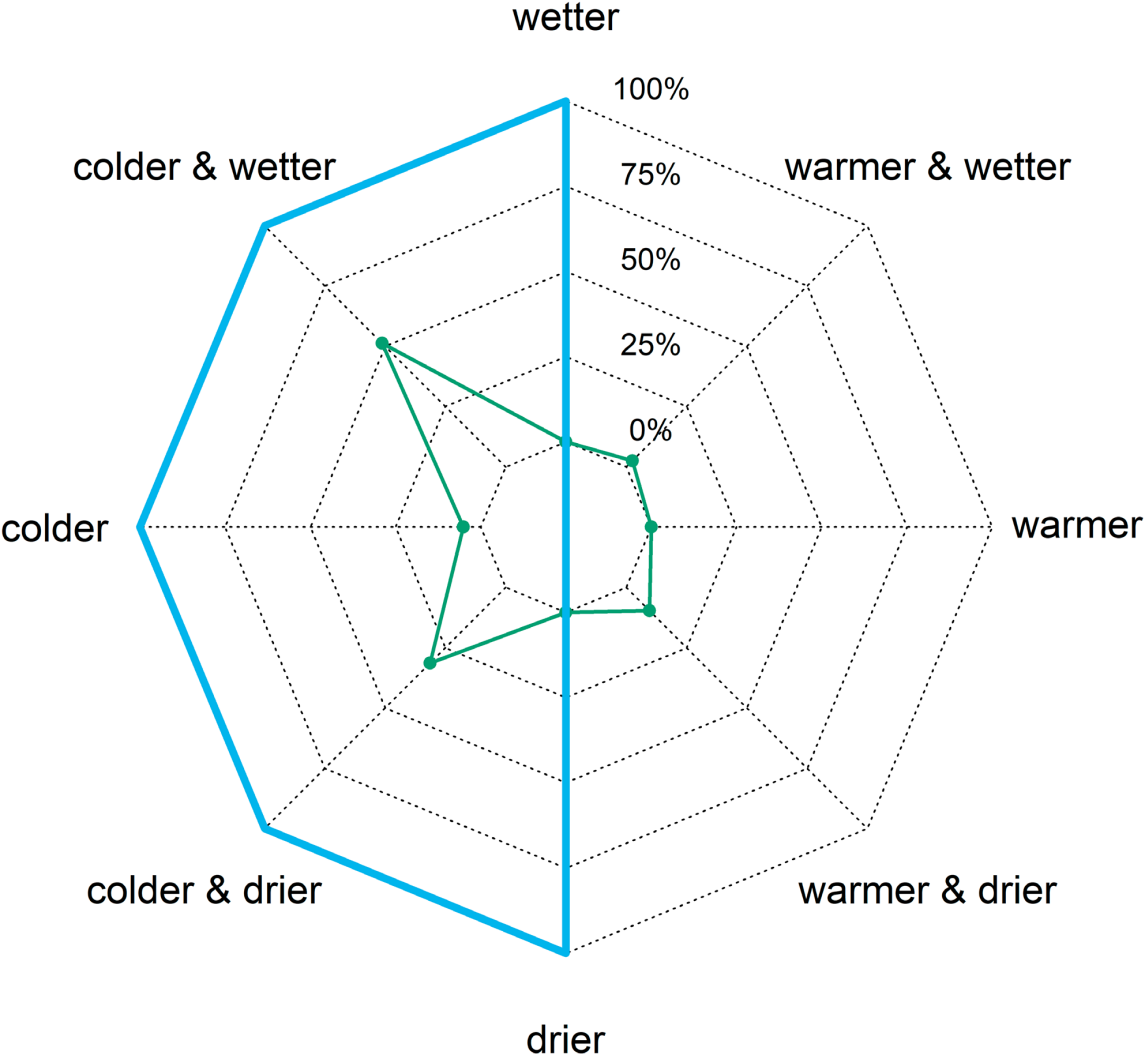
The conditions with highest relative abundances predicted by the spatial climate component relative to temperature and precipitation for the 39 bird species breeding in the Fennoscandian mountains. The green polygon shows the percentages of species for which the spatial climate component predicted highest abundances in locations with different climates. The majority of species had highest predicted abundances in locations that are on average colder, and we focused on these 33/39 species (within the blue polygon). Only six species had highest predicted abundances in locations that are on average warmer (outside the blue polygon) and these six species were not considered for the analysis of the temporal and residual climate components. For most species, the highest predicted abundances fell on only one of the eight climate axes of the spider graph. For the few species which had highest predicted abundances on two of the eight climate directions, we split their contributions in the spider graph proportionally to their distribution of highest predicted abundances (50:50 or 75:25).

Temporal cross‐validation demonstrated that our models were able to generalize to held out years for most species. The Pearson's correlation coefficient between observed and predicted relative abundances was 0.66 (median, range: 0.27–0.83, climate data of the current year), indicating that predicted abundances for most species in held out years had an intermediate or high level of correlation with observed counts. Models based on the same climate data retained the spatial climate component after variable selection for all 39 species. As might be expected for birds breeding in mountain habitats, most species were more abundant at colder sites, demonstrated by a negative association between abundances and long‐term average temperatures (spatial component, Figure 5; Table S3; Figure S8). Only six species (15%) had their highest predicted abundance in locations with warmer long‐term average temperatures (Figure 5): mallard (*Anas platyrhynchos*), common gull (*Larus canus*), Northern raven, common redpoll, fieldfare (*Turdus pilaris*), and willow warbler. These six species are widespread and abundant in Fennoscandia and were not restricted to the mountain region. Thus, we opted to focus on the subset of 33 species that had their highest predicted abundances in colder locations for the spatial component as a common baseline against which to compare associations between species abundances with the temporal and residual climate components.

The spatial component for temperature was always retained in variable selection for each of the 33 species, regardless of whether the climate data were summarized for the current or the previous breeding season (Table 1). Furthermore, the spatial component for precipitation was retained for 31/33 (94%) species. The temporal components for either temperature, precipitation, or both variables were retained for most species (82–88% of species). The spatiotemporal residual components for either temperature, precipitation, or both were less frequently retained, but were still retained for most species (73%–76%, Table 1).

**TABLE 1:** Retention of the spatial, temporal, and residual climate components for the climate variables, temperature, and precipitation in models for 33 species with climate data summarized within either May–July of the current year or the previous year

Climate component	Current year	Previous year
	Number of species/%	Number of species/%
Temperature, precipitation or both		
Spatial	33/100	33/100
Temporal	29/88	27/82
Residual	25/76	24/73
Temperature		
Spatial	33/100	33/100
Temporal	28/85	19/58
Residual	20/61	18/55
Precipitation		
Spatial	31/94	31/94
Temporal	28/85	25/76
Residual	23/70	19/58

**FIGURE 6:**
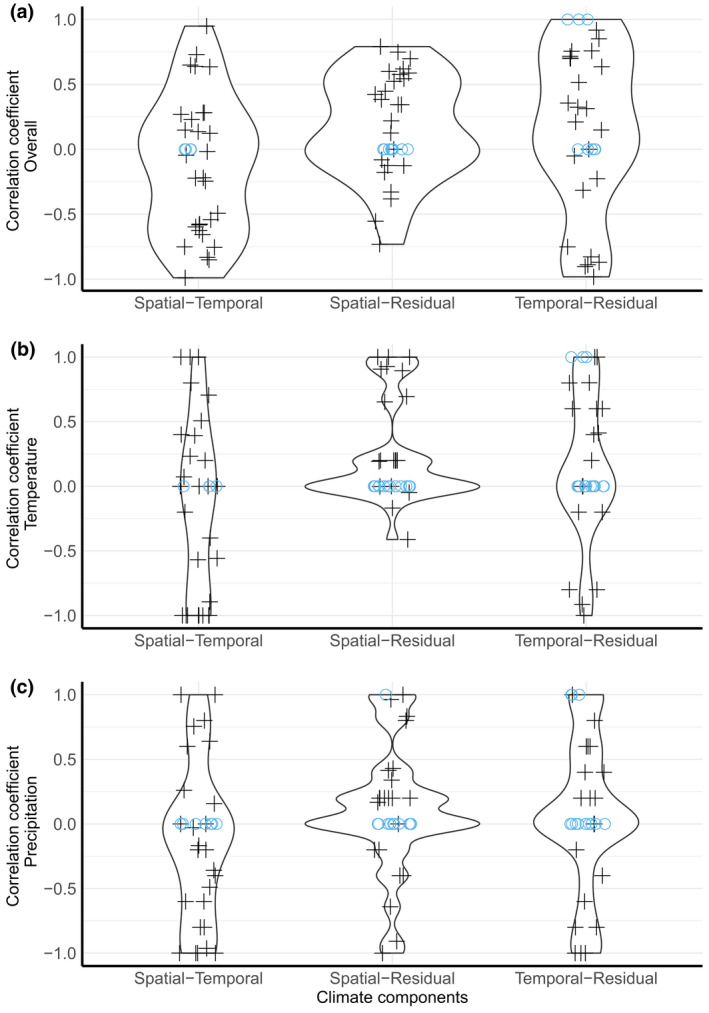
Correlations between the abundances predicted from any two climate components (spatial, temporal, and residual) indicate that the direction of effect was typically not consistent between any two climate components. Violin plots of rank correlation coefficients of pair‐wise comparisons between predicted local species abundances based on the spatial versus temporal climate component, the spatial versus residual climate components, and the temporal versus residual components for (a) temperature and precipitation combined, (b) temperature, and (c) precipitation. Rank correlation coefficients for models with decomposition data from May–July of the current year are shown. For a graphical description of how correlation coefficients were calculated, see Figure S6. Violin plots show the probability density of the data at different values of the correlation coefficient. Black crosses and blue circles represent individual species. Blue circles with correlation coefficients of zero represent species for which no association between a species' local abundance and one of the climate components was found, while an association was found with the other. Blue circles with correlation coefficients of one represent species for which no association was found between the local abundance of the species and either of the climate components. Thus, there is an agreement in conclusions of no effect of either climate component. Crosses represent species for which associations between the local abundance of the species and both climate components were detected. High correlation coefficients indicate that predicted local abundances vary with the climate variable (temperature, precipitation, or both) in the same direction for both climate components (spatial‐temporal, spatial‐residual, or temporal‐residual). Low correlation coefficients indicate that predicted local abundances vary with the climate variable in the opposite direction, for example, local abundances may decrease with temperature for the spatial climate component but increase with the temporal climate component. Correlation coefficients at or near zero indicate that no association was found between the local abundance of the species and one climate component (blue circles) or that associations were complex, such as an increase of predicted local abundance with temperature at low precipitation, but a decrease at high precipitation (Figure S6).

The decomposition method allowed us to separate the spatial and temporal climate components associated with species abundance. Higher abundances of these 33 species were associated with colder locations in the spatial component, but associations with the temporal component were more variable (Figure 6). For these species, higher local abundances were associated with colder places, but not necessarily with colder years. Rank correlation coefficients between the spatial and temporal components were high for only a few species whether temperature and precipitation were considered together, or each climate variable on its own (Figure 6). Negative rank correlation coefficients indicated that the direction of effect between the spatial and temporal components was even opposite for some species. For several species, correlation coefficients were zero or near zero, indicating either that an association between climate and the species' local abundance was only found for one component, or that associations for at least one climate component were more complex, such as an association with lower temperatures when precipitation was high, or conversely with higher temperatures when precipitation was low (Figures 6; Figures S6 and S8; Table S3).

The decomposition method also allowed us to separate the residual from the spatial and temporal climate components. The residual component describes the potential interaction between the spatial and temporal components, such as an annually increasing temperature varying with space. The climate conditions in which the highest local abundances were predicted with the residual component often did not match the climate conditions producing highest predicted abundances for either the temporal or the spatial climate component (Figure 6). Therefore, for most species, neither the temporal nor the residual climate variation had the same direction of effect on local species abundances as the spatial climate variation. Qualitatively similar results were also obtained for models with climate data from the previous season (Figure S9).

For models with climate data from the current season, the subset of species with highest predicted local abundances both in colder locations and in colder years were common snipe (*Gallinago gallinago*), jack snipe (*Lymnocryptes minimus*), red‐necked phalarope (*Phalaropus lobatus*), white‐throated dipper, and redwing (*Turdus iliacus*). These five species had rank correlation coefficients >0.5 between the spatial and temporal climate components for temperature. Similarly, the subset of species with highest predicted local abundances in colder locations, and in warmer years were dunlin (*Calidris alpina*), whimbrel (*Numenius phaeopus*), long‐tailed skua (*Stercorarius longicaudus*), western yellow wagtail (*Motacilla flava*), northern wheatear (*Oenanthe oenanthe*), and Lapland bunting (*Calcarius lapponicus*). These six species had rank correlation coefficients < −0.5 between the spatial and temporal climate components for temperature. Regression coefficients for the nine climate coefficients were only moderately correlated between models with climate decomposition based on the current year versus the previous year (rank correlation coefficients for full models, median: 0.67, min: −0.30, max: 0.97). Thus, higher local abundances may be associated with certain temperature or precipitation conditions in the current breeding season, but not necessarily with the same climatic conditions in the previous breeding season. Our conclusions were unchanged if models were based on climate components from May–June of the current year (Results S2).

## 4 DISCUSSION

Our results show that avian responses to climatic variation are highly species specific and that common SDM forecasting methods may not sufficiently account for diverse responses. First, in addition to the expected effects of the spatial climate components, both temporal and spatiotemporal climate components were associated with local changes in counts for a majority of bird species. The same pattern was observed whether we considered climate variation based on the current or the previous breeding season. Second, while the majority of the bird species had higher predicted local abundances in areas with colder long‐term average temperature, the direction of the effects of climate variation for each species often differed among the spatial, temporal, and residual components. Therefore, our results suggest that the most widely used form of space‐for‐time forecasting, where average climate is used to predict average responses, will fail to account for dynamic changes in local counts. For space‐for‐time substitution to be valid for predicting the impacts of climate change, a direct linkage is needed between species' responses to spatial and temporal variation in climate. This key assumption was seldom met for our focal bird species at the temporal scales we examined. Thus, for the majority of species considered, useful forecasts based on space‐for‐time substitution cannot be generated for the immediate future. Instead, if space‐for‐time substitution is applicable at all, then it will only be valid for forecast horizons that are both left‐ and right truncated. However, appropriate forecast horizons are rarely considered in forecasts with SDMs and are difficult to ascertain. Our third major result was that our findings applied both for species' responses to variation in temperature, as well as to responses to variation in precipitation. Models of species responses to climate variation often focus on temperature, but our results join previous work in indicating that precipitation is also an important driver (Duclos et al., 2019; Illán et al., 2014; Pearce‐Higgins et al., 2015; Tingley et al., 2012). Species' responses to spatial and temporal variation in climate were rarely equivalent for the species that we examined; nevertheless, we did identify a subset of bird species for which predicted local abundances increased with colder conditions in both space and time. Species with negative associations with the temporal climate components may be among the first to be negatively impacted by climate warming in our study regions, as colder breeding seasons are expected to become rarer in the future (Bärring et al., 2017).

Overall, our results for 33 bird species in Fennoscandia extend earlier work by Oedekoven et al. (2017) for five bird species in the UK in showing that the assumptions of space‐for‐time substitutions were not consistent with the temporal species‐climate relationships indicating that limitations to forecast horizons will often exist. Together, our findings indicate that forecasts of bird species' responses to climate change can be made more consistently reliable in several ways. First, we advocate for the routine use of SDMs with dynamic covariates with appropriately selected descriptors of climatic variation. Second, we argue that the forecasting horizon—for when in the future reliable forecasts can be made—should be routinely considered when interpreting and discussing forecasts. Last, we discuss how decomposition SDMs can facilitate the identification of species that are immediately vulnerable to climate change versus species that are initially resilient.

### 4.1 Species distribution models with static, dynamic, and decomposed covariates

Our results suggest that a more dynamic modeling approach to SDMs may improve on SDMs with static covariates. Accounting simultaneously for the effects of seasonal and annual climate variation, potential interannual to decadal climate cycles and climate warming is important because the use of the average future distribution of a species may not be sufficient for efficient conservation action (Dupont‐Doare & Alagador, 2021). It is also vital to understand the pattern of species distributions in the intervening time, the variation in distribution patterns in response to climate variation at different temporal scales, and the mechanisms causing the variation in distribution pattern. For example, long‐term average conditions were adequate to explain the average distribution and catch of a fish species, but deviations in average catch were best explained by interannual variability of the marine environment (Brodie et al., 2021). For some bird species in the US, SDMs with dynamic covariates described the breeding distributions and their dynamic changes better than SDMs with static covariates (Bateman et al., 2016). Similarly, years with extreme conditions such as drought or cold winters can have a strong impact on species abundances and distributions (Pearce‐Higgins et al., 2015), but may be less well represented when future climate data are averaged over many years for forecasts based on SDMs with static covariates.

Our results suggest that temporal changes in local relative abundances were not uniform in space because the spatiotemporal residual component of climate variation was retained in variable selection for the majority of bird species. Retention of the residual climate component suggests that settlement decisions of individuals were dependent on local conditions in specific years. Thus, naïve extrapolation of responses to climatic variation to new regions should be made with caution.

Our finding that climate conditions of both the previous and the current breeding season can influence local abundances of our focal species is consistent with previous results for abundance of bird species in England (Pearce‐Higgins et al., 2015) and population trends of aerial insectivores in North America (Michel et al., 2021). Strong effects of climate during the current breeding season for generalist species in England were explained by warm‐adapted species either settling further north or becoming more detectable during warmer breeding seasons (Pearce‐Higgins et al., 2015). Species settling in greater or smaller numbers in Fennoscandia depending on climatic conditions could also explain some of the variation in species abundances in our study. Here, we evaluated changes in local abundances allowing for increases in local abundances in some regions and decreases in others. Differences in survey protocols restricted our ability to model the detection process and variation in species detectability could have influenced our results. If variation in detectability had confounded our results, we might expect a similar direction of effect on predicted local abundances between the temporal and spatiotemporal climate components, which is not what we found. Davey et al. (2012) controlled for imperfect detection in a study on British birds and found that species richness increased, and the habitat specialism of bird communities decreased in warmer years, which also suggests that climate of the current year can affect species distribution pattern. Thus, our study joins a growing body of evidence that settlement decisions and abundance of birds are influenced by both recent past and current climate conditions, and thus SDMs with dynamic covariates are likely to outperform SDMs with static covariates for predicting species distributions.

The direction of effects from the temporal and residual climate components sometimes differed between models based on climate from the current breeding season versus the previous breeding season. The same climate variable can have opposing effects on species abundances depending on the time lag between climate and abundance estimates (Elston et al., 2017; Pearce‐Higgins et al., 2015). The influence of the climate of the current breeding season is likely driven by settlement decisions or early abandonment of territories based on current conditions. In contrast, climate conditions of the previous or earlier breeding seasons may influence annual production and recruitment, whereas conditions at staging and wintering areas will influence survival of all age classes and the number of individuals available to settle in the current breeding season (Jørgensen et al., 2016; Pearce‐Higgins et al., 2015; Sanderson et al., 2006). Past climatic conditions may also affect settlement decisions if sites with past reproductive success are preferentially occupied (Doligez et al., 2004; Shitikov et al., 2015). Such multiple temporal and spatiotemporal effects of climate are unlikely to be fully accounted for by using year and site‐specific climate covariates. Decomposition SDMs offer a promising way forward to make SDMs dynamic while accounting for the complex spatiotemporal patterns and multiple temporal effects. For example, the temporal component in our models for the previous or current year could be replaced by a more complex temporal component that accounts for lag effects of climate from multiple previous seasons and the current season (Elston et al., 2017). A limitation is that climate data for future scenarios are only available as long‐term averages. However, it may be possible to simulate realistic pattern of annual or seasonal variation for future climate scenarios, which could be used to estimate the range of variability in dynamic forecasts.

### 4.2 Forecasting horizons

For most of our focal species, the assumption of equivalent pattern in space and time was not met for the most general form of space‐for‐time substitution where forecast horizons are truncated only to the right when the modeled species‐climate relationships change (Figures 2 and 3). The spatial climate component did not describe the variation in local annual abundances 1996–2018 and the direction of effects of climate variation differed between space and time. If space‐for‐time substitution based on the spatial component is valid, it is only for static forecasts and for forecast horizons that are left‐ and right truncated. Forecasting under changing species‐climate relationships is currently beyond the purpose and capability of correlative SDMs, and the potential of species‐climate relationships to change via evolutionary adaptation or changes in species interactions has wider implications for right truncation of the forecast horizon (Araujo & Peterson, 2012; Dormann, 2007; Pearson & Dawson, 2003; Singer et al., 2016; Urban et al., 2016). Appropriate time thresholds for left truncation (Figures 2 and 3), where delays of species responses occur, will be difficult to identify for several reasons. First, climate variation affects multiple ecological processes simultaneously and at different temporal scales (Damgaard, 2019). For example, most of 13 forest birds were affected by the direct effects of climate, and by the indirect effects of climate on forest structure and composition, with effects manifesting themselves at different temporal scales (Duclos et al., 2019). Additional factors, such as predation pressure may change at yet other temporal scales and may also interact synergistically with habitat structure (Kubelka et al., 2022; Layton‐Matthews et al., 2020). Second, even for a single ecological process, it may be difficult to accurately forecast when and where the impacts on species distributions will be manifested. For example, succession of alpine mountain habitats to forests may lag behind climate change and can be affected by topographic conditions or land use practices, such as grazing (Bryn, 2008; Kullman, 2001; Wang et al., 2017). Accurately predicting the future distribution of habitats is therefore difficult, even more so when fine thematic and spatial resolutions are needed (Prestele et al., 2016). Similarly, invertebrates are an important food source for many bird species but may be less available in both cold and hot/dry conditions (Barras et al., 2021; Curry, 2004; Pearce‐Higgins, 2010; Pearce‐Higgins & Yalden, 2004; Perez et al., 2016). However, the data are rarely available to determine optima where initially positive effects of warmer conditions on food availability transition into negative effects of hot conditions.

### 4.3 Immediately vulnerable versus initially resilient to climate warming

An important result of our decomposition SDM was identification of a subset of five bird species which may be among the first to be negatively impacted by climate warming. Based on climate data from the current breeding season, predicted local abundances increased with colder conditions in both space and time and the birds were mainly species associated with freshwater habitats (red‐necked phalarope, white‐throated dipper) or inundated areas (common snipe, jack snipe). A possible mechanism for an immediate positive effect of colder years for these species may be through the patterns of snow melt and water levels, with spring floods typically less intense in colder years, while water from snow melt continues to be available for longer. For example, common snipe are dependent on wet soil for foraging, which remain suitable for probing throughout the breeding season, while spring floods may be detrimental for early nesting (Green, 1988). Conversely, based on climate data from the current breeding season, the six species with highest predicted local abundances in colder locations but also warmer years may be initially resilient to a warming climate. Many of these bird species depend on terrestrial invertebrates, at least during the breeding season (dunlin, whimbrel, western yellow wagtail, northern wheatear, and Lapland bunting). A possible mechanism for a potential immediate negative effect of colder years on these species may be a reduction in invertebrates in the Fennoscandian mountains in colder breeding seasons, leading to individuals not settling to breed or prematurely abandoning their territories. A more comprehensive assessment of species that are immediately vulnerable and species that are initially resilient to climate warming would require taking into account additional effects of the climate of previous breeding, migration, and wintering seasons. Multiple climatic effects from different seasons could enhance or counteract each other. Accounting for lag effects would require replacing the temporal component in our decomposition SDMs with a more complex component that includes multiple years (Elston et al., 2017).

For longer forecast horizons in a warming world, the decreases in local abundance that would be predicted by space‐for‐time substitution are realistic for many of our focal species, including birds dependent on open mountain habitats. Even though climate warming may not initially be detrimental to many of these species, in the long‐term, strong negative effects are expected where open mountain habitats are replaced by forests through rising tree lines, or through other factors, such as increased predation from expanding populations of generalist species. However, forecasts are now needed that go beyond correctly forecasting the broad direction of change, but that can accurately forecast change at a fine resolution in both space and time.

In our study area, a landscape monitoring program in Sweden found no change in the extent of the alpine or mountain birch forest areas between the periods 2003–2007 and 2008–2012 (Hedenås et al., 2016). Over longer timescales, tree line rises have mainly been confined to wind‐sheltered and snow‐rich areas in the Swedish mountains (Kullman, 2001). In Norway, upper altitudinal limits of forests have raised during previous decades, mainly driven by regrowth of woody plants after cessation of livestock grazing (Bryn, 2008). Our results suggest that expansion of forests is not yet a primary driver of abundance changes in bird species breeding in the Fennoscandian mountains over large spatial extents as for most species, the direction of effect of climate variation was not consistent in time and space.

## 5 CONCLUDING REMARKS

SDM forecasts with static covariates and space‐for‐time substitution have accurately predicted future species' distribution for some species, but have performed poorly for others (Araujo et al., 2005; Kharouba et al., 2009; Pearman et al., 2008; Soultan et al., 2022). Moreover, even for SDMs that accurately predicted future species distributions, prediction accuracies for sites at which distribution changes occurred were often low suggesting that improvements to forecasting based on SDMs are needed (Briscoe et al., 2021; Illán et al., 2014; Rapacciuolo et al., 2012). Our models with climate variation decomposed into a spatial, temporal, and residual spatiotemporal component revealed that climate variation from both the current and previous breeding season affected local abundances and that species‐climate relationships were equivalent in space and time for only a few species. Our results suggest that forecasts based on SDMs can be improved by (1) making SDMs more dynamic so that forecasts can be produced at the finer temporal resolutions, such as those required for spatial conservation planning (Dupont‐Doare & Alagador, 2021) and (2) obtaining a better understanding of the time spans over which drivers of changes in species distributions and abundances are expected to act. Our results indicate that more dynamic SDMs need to consider spatiotemporal variation in addition to multiple temporal effects of climate variation. Models that integrate extensive occurrence or abundance data from surveys that cover large areas with intensive demographic data collected in smaller areas where patterns of species distributions emerge from population dynamics should help with a better understanding of the temporal scales over which ecological processes act (Zipkin et al., 2021). However, the comprehensive population data needed to parameterize alternative models including population dynamics is available for few species (Bradter et al., 2021; Urban et al., 2016), suggesting that correlative SDMs will remain important as they can be parameterized with more widely available data. Our results suggest that SDMs based on a decomposition of covariates can increase our understanding of species responses to climatic variation and that caution is required when using space‐for‐time substitutions based on correlative SDMs.

## CONFLICT OF INTEREST

All authors declare no conflict of interest.

## Supporting information


Appendix S1

## Data Availability

The bird survey data are available at GBIF: www.gbif.org/dataset/4a00502d‐6342‐4294‐aad1‐9727e5c24041 (Norway), www.gbif.org/dataset/91fa1a0d‐a208‐40aa‐8a6e‐f2c0beb9b253 (Sweden), www.gbif.org/dataset/961d16be‐525e‐47b8‐bc96‐077df8224ae0 (Finland). Information for sensitive species and sites has been redacted to ensure protection from unlawful use. Complete datasets are available from the program coordinators for approved projects. The Nordic Gridded Climate Data (NGCD) are available from thredds.met.no/thredds/catalog/ngcd/catalog.html. CORINE land cover data are available from: land.copernicus.eu/pan‐european/corine‐land‐cover. The digital elevation map (DEM) was obtained from land.copernicus.eu/imagery‐in‐situ/eu‐dem/eu‐dem‐v1.1. R scripts for our analysis are available at https://github.com/UteBradter.
