# Supplementary material for: Decomposing the spatial and temporal effects of climate on bird populations in northern European mountains
Source: Glob Chang Biol. 2022 Aug 14;28(21):6209–27. doi: 10.1111/gcb.16355 (PMC9804621; doi:10.1111/gcb.16355)
Supplement: Supplementary file 1 — Appendix S1 [file GCB-28-6209-s001.docx]

## Supporting information

for:

Ute Bradter, Alison Johnston, Wesley M. Hochachka, Alaaeldin Soultan, Jon E. Brommer, Elie Gaget, John Atle Kålas, Aleksi Lehikoinen, Åke Lindström, Sirke Piirainen, Diego Pavón-Jordán_,_ Tomas Pärt_,_ Ingar Jostein Øien & Brett K. Sandercock (2022) Decomposing the spatial and temporal effects of climate on bird populations in northern European mountains. Global Change Biology.

## S1: Methods

### Figs 2, 3

The temperatures shown in Fig. 2a, Period 1, are the average of the temperatures in Year 1-6 in Fig. 3a. Periods 2-6 in Fig. 2a represent periods after a warming of 2°C in each of the six years of the period has occurred.

For slow responses, the simulated abundances were calculated as log(*α* + *ß_1_* * *Space_i_*), where *Space_i_* is the long-term average temperature at each location *i* in a period*.* For fast responses, the simulated abundances were calculated as log(*α* + *ß_1_* * *Temp_i, t_*)*_,_* where *Temp_i, t_* is the temperature at each location *i* and year *t*. For species with mixed responses to temperature variation, the simulated abundances were calculated as log(*α* + *ß_1_* * *Space_i_* + *ß_2_* * *TempDiff_i, t_*), where *TempDiff_i, t_* is the temperature difference at each location *i* in year *t* to the long-term average temperature *Space_i_*. The coefficients were *α* = 0, *ß_1_* = 0.9, *ß_2_* = -0.3.

For simulated abundances within a six year period, we averaged the simulated abundances across the six years (median). For simulated abundances of virtual species experiencing a delay in their response to changes in the long-term average temperature, Period 1 was used until the delay was overcome.

Applying SDMs with static covariates (summarizing temperatures within six-year periods) to each of the virtual species would estimate *ß_1_≈ 0.9* for the virtual species in both Fig. 2 and Fig. 3. Consequently, forecasts based on space-for-time substitution do not differ between the virtual species.

### Bird monitoring data

In Sweden and Finland, survey routes were deployed on a regular grid of 25 km × 25 km and in Norway on a subsample of a regular 18 km × 18 km grid. In all three countries, southern areas and lowlands were surveyed earlier in the breeding period while northern areas and mountains were surveyed later to coincide with the peak of the breeding bird activity. Surveys were carried out early morning and in good weather conditions (no precipitation or strong wind). Birds were detected by both auditory and visual cues by trained observers using standardized field protocols that specified when during the breeding season a route should be surveyed, the time of day and weather conditions during which surveys could be carried out, the approximate walking speed for transect surveys, the observation period for point count surveys (5 min) and how to record the birds observed. Field protocols are available at [tov-e.nina.no/Fugl/public/papirskjema/MethodologyEng.pdf?ver=1](https://tov-e.nina.no/Fugl/public/papirskjema/MethodologyEng.pdf?ver=1) for Norway, [www.fageltaxering.lu.se/inventera/metoder/standardrutter/metodik-standardrutter](http://www.fageltaxering.lu.se/inventera/metoder/standardrutter/metodik-standardrutter) for Sweden and [www.luomus.fi/en/methods-bird-monitoring](http://www.luomus.fi/en/methods-bird-monitoring) for Finland. Observations of flocks of birds were recalculated to the number of pairs (for Norway and Finland) by dividing the number of individuals in the flock by two.

### Environmental data

Slope and incoming solar radiation were calculated from the digital elevation model (DEM). Radiation was calculated in ArcGIS 10.7.1 (ESRI, 2010) as a function of topography and latitude using the Area Solar Radiation tool and the DEM resampled to a coarser resolution of 200 m.

Of the seven land cover covariates, we always included Wetlands and Water, as these are important for many species. As the number of covariates in our models was already relatively large, we added additional land cover categories depending on each species. For species listed as occurring in mountains, but not forests according to Artfakta (see main text: we used species accounts from Artfakta of Sweden, as the central country of our study area), we included the two mountain categories Sparsely Vegetated Mountain Areas and Mountain Vegetation. For species listed as occurring in both mountains and forests, we selected a subset of the four mountain and forest land cover categories according to species habitat descriptions in Svensson et al. (2009), which describes a finer thematic resolution compared to the coarse habitat groupings of Artfakta. For species occurring in agricultural landscapes we added Agriculture. For a subset of selected species, we fitted solar radiation or quadratic functions for Water, Wetlands and Agriculture, to test the possibility that solar radiation influences local relative abundances or that intermediate levels of different land cover categories may be beneficial for the species. For regression coefficients of land cover coefficients, see Table S2.

### Model selection with AIC

We performed model selection by removing covariates in the following order: (1) quadratic terms of second order polynomials, (2) residual climate interaction between temperature and precipitation, (3) temporal climate interaction between temperature and precipitation, (4) spatial climate interaction between temperature and precipitation, (5) year, (6) residual climate components, (7) temporal climate components, (8) spatial climate components, (9) topographical covariates, (10) Agriculture, (11) Water, (12) Wetland, (13) Forests, (14) Mountains. If the AIC of the reduced model was lower than the AIC of the model before the covariate’s removal, the covariate was permanently removed, otherwise it was added back in. We evaluated the climate decomposition components before topographical and land cover covariates, because the focus of our models was on the potential effects of climate variation. Therefore, we evaluated the climate covariates while controlling for topography and land cover and only thereafter evaluated topographical and land cover covariates.

If applicable, each covariate was removed first in the zero-inflation part of the model. For the spatial, temporal, and residual climate components, Forests and Mountains, which consisted of two covariates each, we compared the AICs from models with both, a single, or neither of the two covariates and selected the model with the lowest AIC.

### Assessing the effects of the climate components

When we predicted from the models, we fixed non-climate covariates to the following values typical for the data set: we retained non-climate covariates for all 1749 routes with line transects, selecting the most recent land cover information and setting the continuous covariate ‘Year’ to the mean value. We set the survey specific covariates to typical values for Sweden (Unit: Individuals; Effort: 8 km, the maximum length of fixed routes in Sweden).

### Cross-validation

In the cross-validation procedure, we withheld data for one year, fitted the model on the remaining data and then predicted avian abundance for the withheld year. We repeated these steps until each of the 23 years 1996–2018 were withheld once. Then we calculated the Pearson correlation coefficient between all observed and all predicted values across all routes.

### Robustness tests

We assessed the robustness of our models to spatial and temporal sample selection bias in the monitoring data (tests 1 and 2 below) and to the joint analysis of data from three national monitoring schemes, with differences in national survey protocols (test 2 below). We assessed the robustness of our models by checking qualitative agreement in regression coefficients when refitting the models for each species to two subsets of the data, each with a different spatial and temporal bias. First, we refitted each model to data for a shorter time period (2007 – 2018), where geographic coverage was more complete compared to the full dataset. Second, we refitted each model to data from Sweden only which had the longest time series for the standardized breeding surveys. In addition to changing the spatial and temporal bias in the data, our validation procedures allowed us to verify the robustness of our joint analysis of data from national survey schemes with different protocols. Some differences in estimates of regression coefficients and confidence intervals may occur when a model is fitted to different samples of data. Differences in estimation of regression coefficients and confidence intervals may also occur when the range of variation of covariates differs between subsets of data. Our samples of data contained difference in range of variation of covariates, particularly for topographical and land cover covariates, and particularly when we compared models with data from Sweden versus models with data from the much larger extent covering all three countries. Therefore, we concentrated our assessment of a qualitative agreement in regression coefficient on the temporal and residual climate components, where the range of variation was less dependent on the spatial extent of the data (see Fig. 4 in main text). We considered models to qualitatively agree, if regression coefficients with 95% confidence intervals that did not overlap zero retained the same sign and a broadly similar magnitude. Additionally, we assessed the possible influence of residual autocorrelation on our model results (test 3 below). The robustness tests were based on the data set with the climate data from May-July of the current year.

1. Data from 1996-2018 versus data from 2007-2018

We compared regression coefficients from the models with all data (1996-2018) to regression coefficients from models with data from 2007-2018 where geographic coverage was more complete compared to the full dataset and spatial and temporal sample selection bias was therefore reduced. Regression coefficients from the models with all data (Norway and Finland: 2006-2018, Sweden: 1996-2018) for the temporal and residual climate components, and mostly also for other environmental covariates, were qualitatively in good agreement with regression coefficients from the models with data from 2007-2018, suggesting that conclusions from the models were robust to the spatial and temporal sample selection bias in the data (Fig. S1). The only exception was common redpoll for which the regression coefficients for the temporal climate components changed sign between the two models. However, common redpoll was one of six species for which we only present associations with the spatial climate component, but not with the temporal and residual climate components.


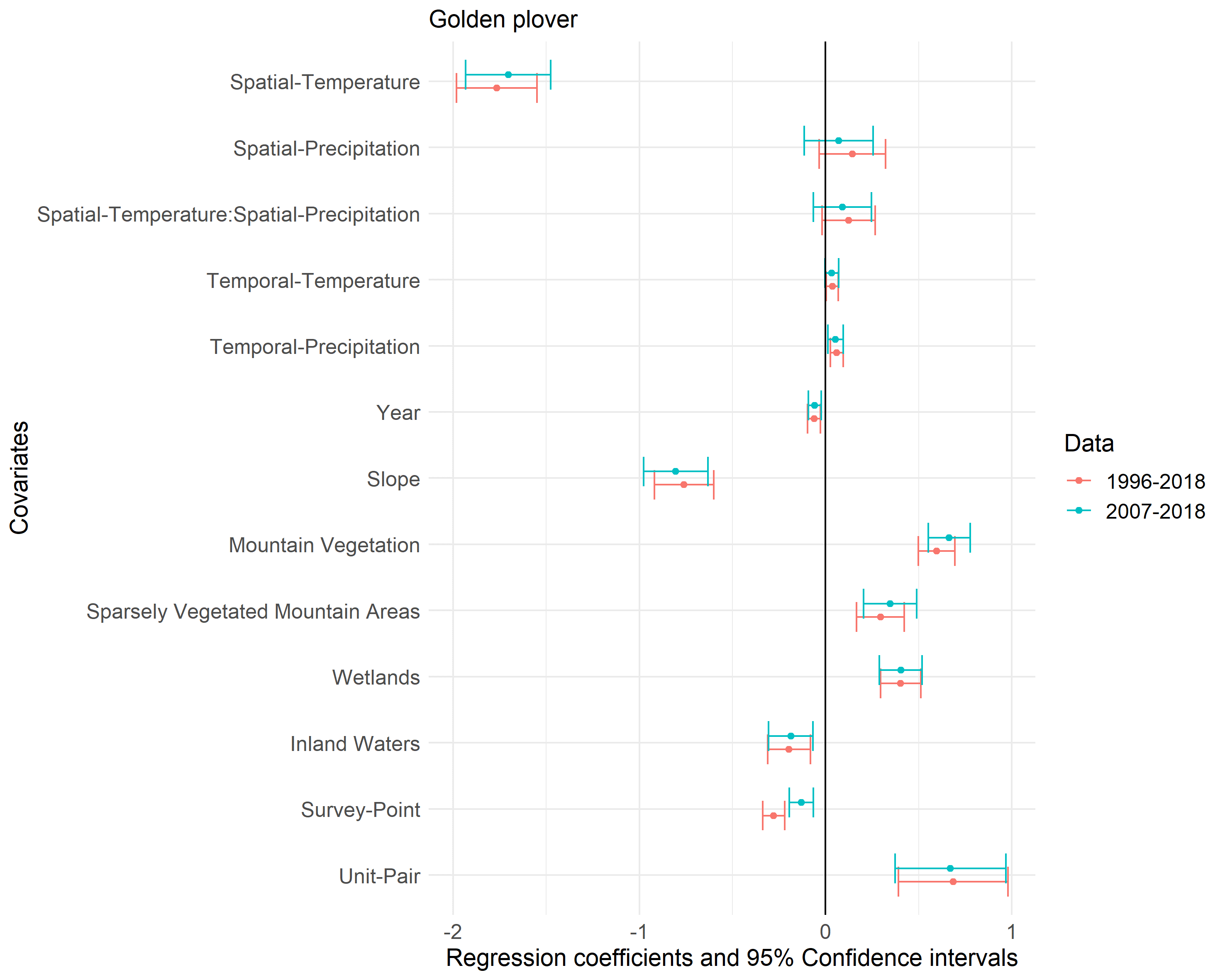


#### Fig. S1: Regression coefficients from models with all data (1996-2018) and from models with data from a shorter period (2007-2018) were qualitatively similar.

The plot shows an example for one species, golden plover (*Pluvialis apricaria*). An interaction between covariates is indicated by ‘:’. The levels of the two categorical covariates Survey and Unit are given after ‘-‘.

1. Data from Fennoscandia versus data from Sweden only

We compared regression coefficients from the models with all data from Fennoscandia to regression coefficients from models with data from Sweden only. In addition to changing the spatial and temporal bias in the data, our validation procedures allowed us to verify the robustness of our joint analysis of data from national survey schemes with different protocols. Discrepancies in regression coefficients for some covariates might be expected due to the larger range of variation in slope and land cover in the full dataset compared to the Sweden-only data since the majority of the mountain areas are located in Norway. Thus, a divergence may not indicate an effect of spatial or temporal sample selection bias. Differences in regression coefficients for the zero-inflation part of the model were also expected because the Sweden-only dataset is smaller and will contain fewer excess zeros where zeros can be due to surveys in areas with suitable habitat, but at the edge or outside of the range of a species, which may be occupied in fewer years, or not at all. Consequently, we assessed the qualitative agreement in regression coefficients for the temporal and residual climate components in the count part of the model. Regression coefficients for the temporal and residual climate components from the count part of the models with all data were overall in good agreement with regression coefficients from the models with data from Sweden only (Fig. S2).

The consistency of the regression coefficients from the climate components in the count part of the models, when data from all three countries or only from Sweden were used, suggests that the conclusions from our models are robust to the joint analysis of the data despite the differences in the survey protocols among the three countries. The consistency of regression coefficients for the climate components in both robustness tests (1 and 2) suggest that model conclusions were robust to spatial and temporal sample selection bias.


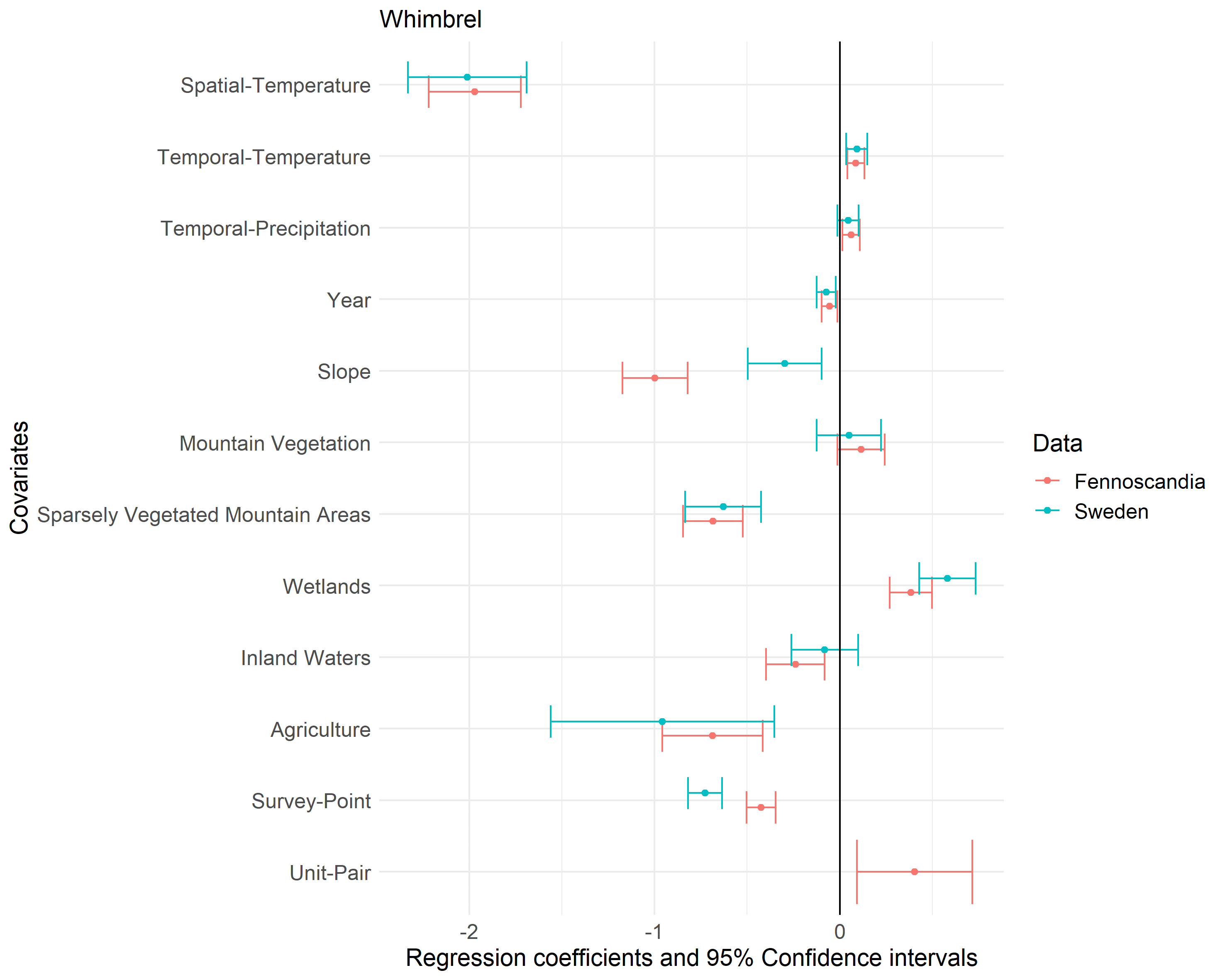


#### ***Fig. S2: Regression coefficients for climate components from models for Fennoscandia and from models for Sweden were qualitatively similar.***

The plot shows an example for one species, whimbrel (*Numenius phaeopus*). The levels of the two categorical covariates Survey and Unit are given after ‘-‘.

1. Residual autocorrelation

To assess residual temporal autocorrelation we carried out a Durbin-Watson test on the residuals of each route. We applied a significance threshold of alpha = 0.05 and a Bonferroni correction due to the multiple testing of residuals from multiple routes. To assess residual spatial autocorrelation we calculated Moran’s I autocorrelation coefficient for the model residuals for each year. As the number of data points was large (1193 point count stations and 1749 line transects in total), we used a threshold of alpha = 0.001 for significance testing and applied a Bonferroni correction due to the multiple testing of residuals in multiple years. Of the 33 species for which result for all three climate components were presented (main text), only 8 species retained temporal residual autocorrelation in a small percentage of routes (< 0.1% of routes). Two species (common cuckoo, redwing) retained residual spatial autocorrelation in each year, while a further three species retained residual spatial autocorrelation in over 50% of the years (whinchat, common snipe, Western yellow wagtail, Fig. S3). Plots of residuals in geographic space revealed that residual spatial autocorrelation was at least partly related to the differences in survey protocols, as residual pattern differed between countries for several species. Therefore, while regression coefficients of environmental covariates in models appeared to be robust to the joint analysis of data from the three different survey schemes (see robustness tests above), the three covariates Effort (length of transect lines or number of point count stations), Survey (line transect or point counts) and Unit (individuals or pairs) may not always have fully accounted for differences in survey protocols. We could have attempted to eliminate the remaining spatial structure in the residuals for example by fitting spatial eigenvectors from a principal coordinate analysis of neighbour matrices or using Gaussian Markow random fields (Dray et al., 2006; Zuur et al., 2017). However, our models were already complex, because they included zero-inflation and several terms from the climate decomposition, and we did not attempt these methods to avoid problems with model convergence.

Residual spatial autocorrelation can increase Type I error rates (Dormann et al., 2007) and for the species with a high proportion of years with residual spatial autocorrelation we cannot rule out that Type I error was increased. However, the number of climate components selected in models was not explained by the proportion of years with significant residual spatial autocorrelation in model residuals (generalized linear models with a Poisson distribution: p = 0.74 for 16 zero-inflated models and p = 0.63 for 17 models without zero-inflation, Fig. S4).


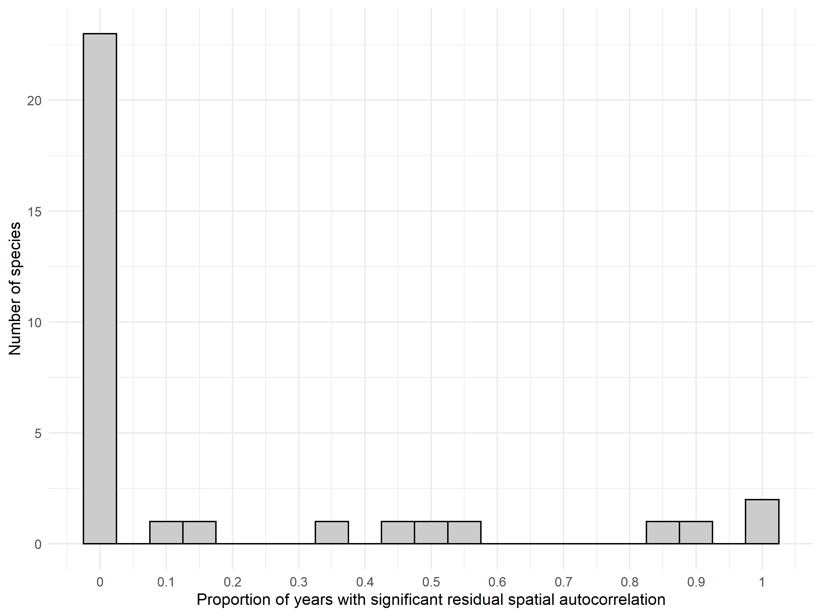


#### Fig. S3: Histogram for final models of 33 species showing the proportion of years with significant residual spatial autocorrelation in the model residuals at a significance threshold of p >= 0.001).


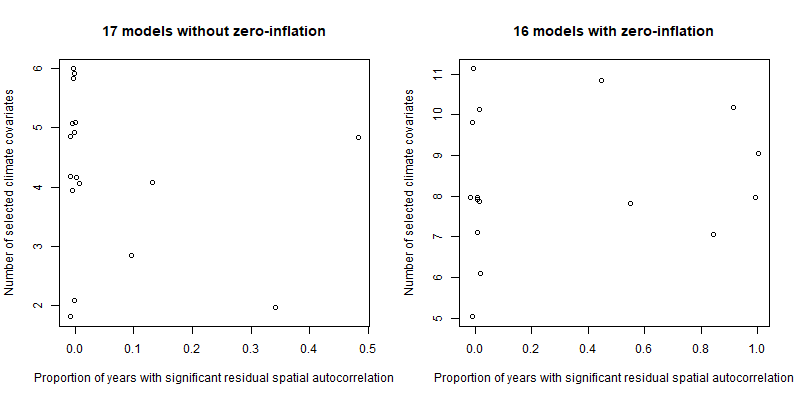


#### Fig. S4: The number of climate covariates retained in final models was not associated with the proportion of years with significant spatial autocorrelation of residuals.

Left: 17 models without zero-inflation and right: 16 models with zero-inflation. The final models for six additional species for which only the results of the spatial climate component were presented in the main manuscript, were not considered.

**References**

Dormann, C. F., McPherson, J. M., Araújo, M. B., Bivand, R., Bolliger, J., Carl, G., . . . Wilson, R. (2007). Methods to account for spatial autocorrelation in the analysis of species distributional data: a review. *Ecography, 30*, 609-628.

Dray, S., Legendre, P., & Peres-Neto, P. R. (2006). Spatial modelling: a comprehensive framework for principal coordinate analysis of neighbour matrices (PCNM). *Ecological Modelling, 196*(3-4), 483-493.

ESRI. (2010). ArcGIS 10..7.1. *ESRI, Redlands, California*.

Svensson, L., Mullarney, K., & Zetterström, D. (2009). *Collins bird guide* (Vol. 2nd edition). London: Harper Collins Publishers.

Zuur, A. F., Ieno, E. N., & Saveliev, A. A. (2017). *Beginner's guide to spatial, temporal and spatial-temporal ecological data analysis with R-INLA*. Newburgh, UK: Highland Statistics Ltd.

## Table S1: The 39 bird species included in this study.

As an index of relative abundance, the number of routes x years with detections of the species is given in column ‘Detections’. The number of routes surveyed per year varied from 96 routes in 1996 to 2002 routes in 2018. During the period 1996 to 2018, on average 1152 routes (mean) were surveyed per year. During the period 2007 to 2018, on average 1715 routes (mean) were surveyed per year.

**English name Scientific name Detections**

Mallard *Anas platyrhynchos*  4021

Tufted duck *Aythya fuligula* 1287

Long-tailed duck *Clangula hyemalis* 283

Red-breasted merganser *Mergus serrator* 955

Willow grouse *Lagopus lagopus* 2311

Rock ptarmigan *Lagopus muta* 1092

Common ringed plover *Charadrius hiaticula* 646

Eurasian dotterel *Charadrius morinellus* 352

European golden plover *Pluvialis apricaria* 4070

Purple sandpiper *Calidris maritima*  80

Ruff *Calidris pugnax* 292

Dunlin *Calidris alpina* 401

Temminck’s stint *Calidris temminckii* 203

Wood sandpiper *Tringa glareola* 5885

Common redshank *Tringa totanus* 2562

Spotted redshank *Tringa erythropus* 541

Whimbrel *Numenius phaeopus* 2863

Common snipe *Gallinago gallinago* 7600

Jack snipe *Lymnocryptes minimus* 284

Red-necked phalarope *Phalaropus lobatus* 262

Long-tailed skua *Stercorarius longicaudus* 672

Common gull *Larus canus* 9295

Arctic tern *Sterna paradisaea* 1097

Common cuckoo *Cuculus canorus* 17929

Meadow pipit *Anthus pratensis* 6474

Western yellow wagtail *Motacilla flava* 5030

White-throated dipper *Cinclus cinclus* 276

Bluethroat *Luscinia svecica* 1742

Whinchat *Saxicola rubetra* 7421

Redwing *Turdus iliacus* 15132

Fieldfare *Turdus pilaris* 11019

Ring ouzel *Turdus torquatus* 1137

Northern wheatear *Oenanthe oenanthe* 3741

Willow warbler *Phylloscopus trochilus* 22997

Northern raven *Corvus corax* 8334

Common redpoll *Acanthis flammea* 5904

Reed bunting *Emberiza schoeniclus* 6094

Snow bunting *Plectrophenax nivalis* 546

Lapland bunting Calcarius lapponicus 834


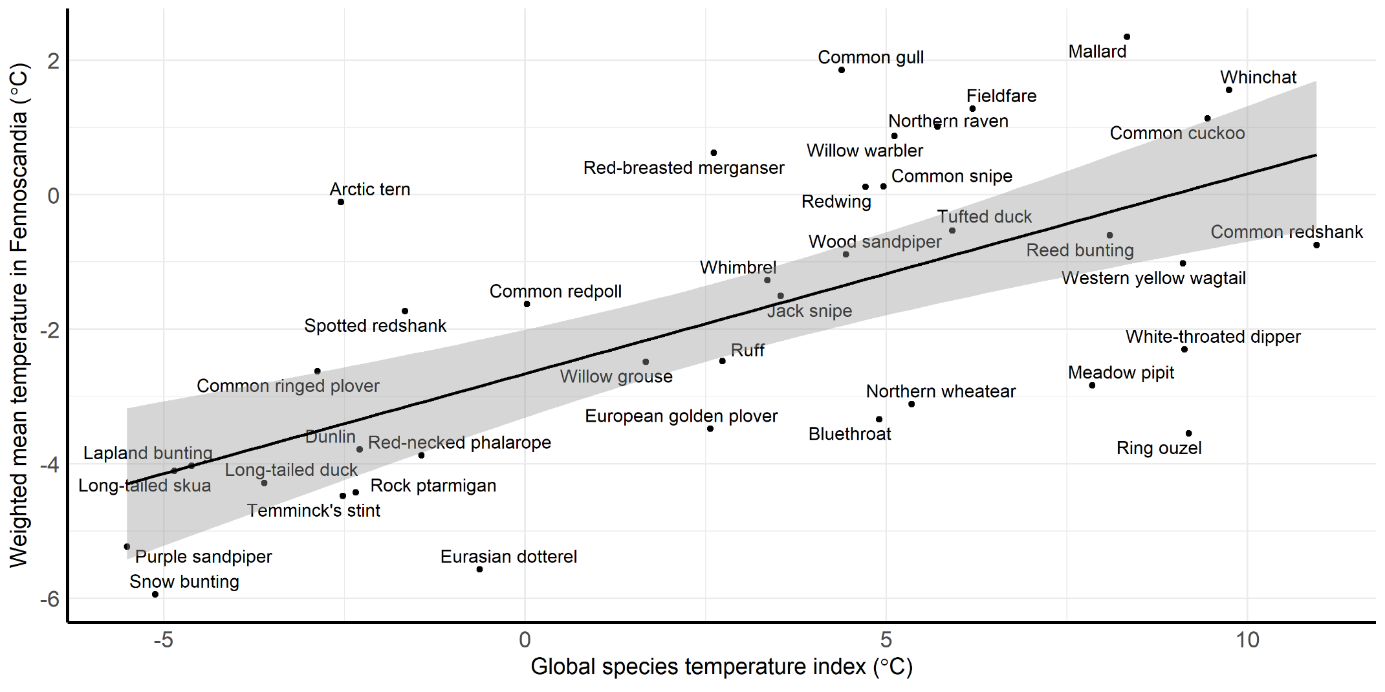


## Fig. S5: The global species temperature index (STI) values of 39 bird species breeding in the Fennoscandian mountains as a predictor of the weighted mean location of each species relative to the centered long-term average breeding season temperature (May-July, 10.7°C) in Fennoscandia (spatial climate component).

The distribution of each species in Fennoscandia relative to temperature was approximately consistent with the global distribution of each species along a global temperature gradient (*Pearson’s r* = 0.65, p < 0.001). The global distribution was represented by the global species temperature index (STI). We computed the STI following Devictor et al. (2008) as the mean long-term average temperature from March to August (WorldClim, 1950-2000, www.worldclim.com) within the global breeding range of a species (BirdLife International and Handbook of the Birds of the World, 2018). To calculate a regional temperature index for each species in Fennoscandia, we predicted the abundance of each species at each survey route. We predicted abundance for routes with line transects using the most recent land cover information and the observed spatial climate components, while setting the continuous covariate ‘Year’ to the mean value, effort to 8 km (the most common value in the data) and the effect of the temporal and residual climate components to zero. For each species, we then calculated their mean temperature weighted by the predicted abundances.

**References**

BirdLife International and Handbook of the Birds of the World. (2018). *Bird species distribution maps of the world*.

Devictor, V., Julliard, R., Couvet, D., & Jiguet, F. (2008). Birds are tracking climate warming, but not fast enough. *Proceedings Of The Royal Society B-Biological Sciences, 275*(1652), 2743-2748. doi:10.1098/rspb.2008.0878


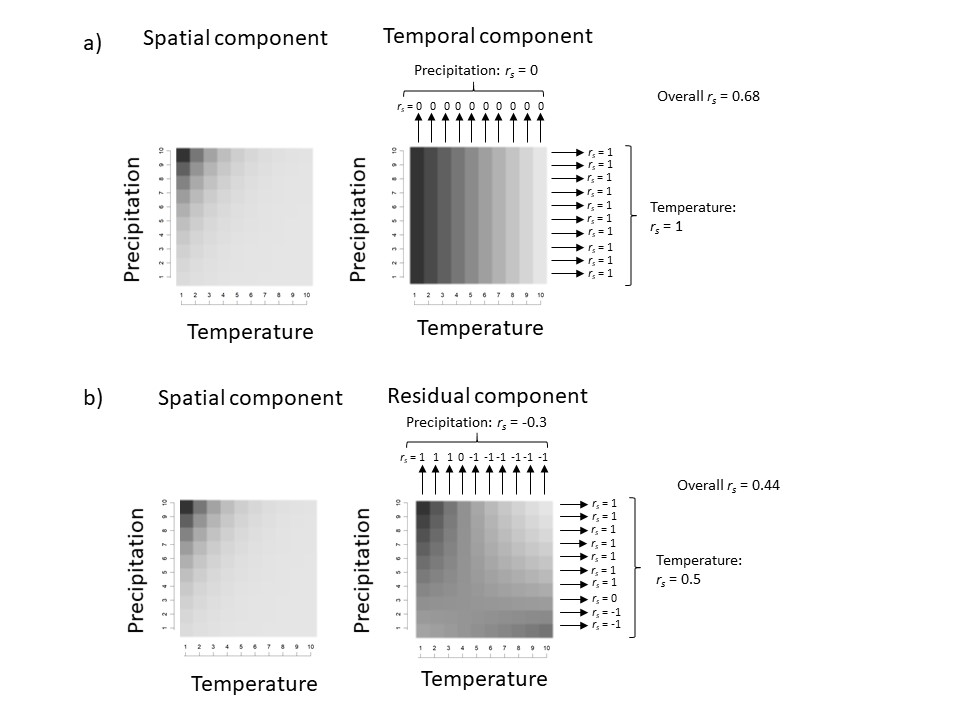


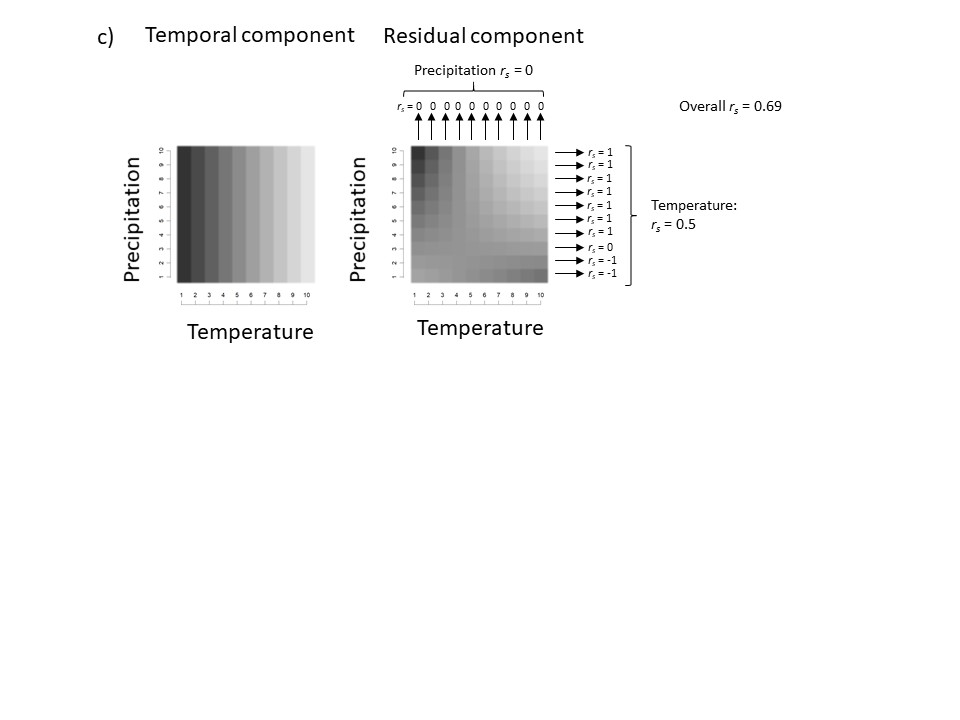


## Fig. S6: Calculation of rank correlation coefficients from predicted relative abundances

With the decomposition SDMs we predicted the relative abundance for each species at each route with line transects while varying temperature and precipitation first for the spatial component, then for the temporal component and finally for the residual component. We varied the temperatures and precipitations at which predictions were made along ten evenly spaced values between the observed minimum and maximum decomposed temperature and precipitation values, while all other covariates were fixed to representative values. Predicted relative abundances were summed across routes for each of the 10 × 10 combinations of temperature with precipitation. Then we calculated Spearman’s rank correlation coefficient (*r_s_)* between predicted relative abundances of a) the spatial and temporal components, b) the spatial and residual components and c) the temporal and residual components. Three sets of correlation coefficients were calculated: (1) an overall correlation coefficient based on all predicted relative abundances, (2) a correlation coefficient for temperature calculated as the mean of ten correlation coefficients, each based on the ten predicted relative abundances for each fixed precipitation value and (3) a correlation coefficient for precipitation calculated as the mean of ten correlation coefficients, each based on the ten predicted relative abundances for each fixed temperature value. If predicted relative abundances for one component did not vary along the temperature gradient, the precipitation gradient, or both, the corresponding correlation coefficient was set to zero, see precipitation in a). If predicted relative abundances for both climate components did not vary along the climate gradients, the corresponding correlation coefficient was set to one to represent the agreement in the conclusion of no effect of climate.

## Table S2: Regression coefficients for land cover covariates

Regression coefficients for land cover covariates for 39 species after variable selection. Species abundances were modelled using a negative bionimal distribution and a log link with or without zero-inflation with the exception of white-throated dipper for which a Poisson distribution was used. For species with zero-inflated models, the table contains separate rows for the count and the zero-inflation part of the model. For species with models without zero-inflation, the table contains one row for the counts only. Covariates which were removed during variable selection are denoted by ‘x’. Water, wetlands, and agriculture were modeled with both linear and quadratic relationships. Regression coefficients from models based on climate data from May-July of the current year are presented. For regression coefficients of climate components, see Table S3.

|  |  |  |  |  |  | **Mountain** | **Sparsely** | **Decidious** | **Other** |  |  |
| --- | --- | --- | --- | --- | --- | --- | --- | --- | --- | --- | --- |
| **Species** | **Part** | **Water^2^** | **Water** | **Wetlands^2^** | **Wetlands** | **vegetation** | **vegetated** | **forest** | **forest** | **Agriculture^2^** | **Agriculture** |
| Mallard | count |  | 0.71 |  | 0.29 | x | x |  |  | -0.15 | 0.94 |
|  | zero |  | 0.43 |  | -0.22 | -0.42 | 0.12 |  |  | x | -0.40 |
| Tufted duck | count |  | 0.56 |  | 0.22 | 0.23 | -0.26 |  |  |  |  |
|  | zero |  | 0.33 |  | x | x | 0.35 |  |  |  |  |
| Long-tailed duck | count |  | x |  | x | 0.65 | x |  |  |  |  |
| Red-breasted merganser | count |  | 0.48 |  | x | x | -0.28 |  |  |  |  |
|  | zero |  | x |  | x | -0.42 | -1.03 |  |  |  |  |
| Willow grouse | count |  | 0.16 |  | 0.57 | 0.44 |  | 0.60 | 0.64 |  | -1.14 |
| Rock ptarmigan | count |  | 0.28 |  | x | 0.32 | 0.54 |  |  |  |  |
|  | zero |  | x |  | 0.53 | -0.61 | -0.45 |  |  |  |  |
| Common ringed plover | count |  | 0.25 |  | x | 0.58 | 0.76 |  |  |  |  |
| Eurasian dotterel | count |  | x |  | x | 0.33 | 0.57 |  |  |  | 0.66 |
| Eurasian golden plover | count |  | -0.20 |  | 0.40 | 0.60 | 0.30 |  |  |  | x |
| Purple sandpiper | count |  | x |  | x | x | 0.62 |  |  |  |  |
| Ruff | count |  | x |  | 0.67 | 0.35 | x |  |  |  | 0.54 |
| Dunlin | count |  | x |  | x | 0.57 | 0.56 |  |  |  |  |
| Temminck's stint | count |  | x |  | x | x | x |  |  |  |  |
|  | zero |  | -0.30 |  | x | -0.28 | -0.89 |  |  |  |  |
| Wood sandpiper | count |  | -0.26 | -0.11 | 0.62 | -0.11 | -0.57 |  |  |  | -0.69 |
| Common redshank | count |  | 0.18 |  | 0.32 | 0.53 | x |  |  |  | 0.57 |
| Spotted redshank | count |  | -0.42 |  | 0.23 | -0.18 | -1.25 |  |  |  | x |
| Whimbrel | count |  | -0.24 |  | 0.38 | 0.11 | -0.68 |  |  |  | -0.69 |
| Common snipe | count |  | x | -0.04 | 0.35 | 0.18 | -0.14 |  |  |  | -0.21 |
|  | zero |  | -0.60 | 0.03 | -0.03 | -0.27 | 0.32 |  |  |  | x |
| Jack snipe | count |  | -1.52 |  | 0.42 | x | -1.05 |  |  |  |  |
| Red-necked phalarope | count |  | x |  | 0.45 | 0.92 | x |  |  |  |  |
| Long-tailed skua | count |  | -0.36 |  | -0.60 | 0.32 | x |  |  |  |  |
|  | zero |  | x |  | -0.74 | -1.45 | -1.31 |  |  |  |  |
| Common gull | count | -0.10 | 0.98 |  | 0.14 | 0.52 | 0.37 |  |  | -0.15 | 0.88 |
|  | zero | x | -0.51 |  | 0.52 | -0.84 | x |  |  | 0.13 | 0.38 |
| Arctic tern | count |  | 0.55 |  | x | 0.32 | -0.40 |  |  |  |  |
|  | zero |  | x |  | x | -0.14 | -0.98 |  |  |  |  |
| Common cuckoo | count |  | -0.07 | -0.05 | 0.22 | 0.18 |  | 0.12 |  | 0.04 | -0.38 |
|  | zero |  | -2.13 | -0.10 | 0.81 | -0.65 |  | -0.13 |  | -0.45 | 1.94 |
| Meadow pipit | count |  | -0.19 |  | 0.47 | 0.67 | 0.29 |  |  |  | 0.68 |
| Western yellow wagtail | count |  | -0.11 |  | 0.39 | -0.39 | -0.91 |  |  |  | 0.26 |
|  | zero |  | -1.63 |  | x | -1.09 | 1.21 |  |  |  | 2.98 |
| White-throated dipper | count |  | x |  | x | -0.23 | -0.30 |  |  |  |  |
| Bluethroat | count |  | 0.15 |  | 0.26 | 0.48 |  | 0.63 |  |  | x |
|  | zero |  | -2.13 |  | -0.22 | -0.87 |  | -0.20 |  |  | 3.69 |
| Whinchat | count |  | -0.16 | x | 0.05 | -0.56 | -0.36 | -0.06 |  | -0.11 | 0.76 |
|  | zero |  | -0.87 | x | x | -1.72 | 1.09 | 0.28 |  | x | 0.36 |
| Redwing | count |  | 0.37 |  | 0.45 | 0.39 |  | 0.58 | 1.17 |  | 0.49 |
|  | zero |  | -1.75 |  | 0.40 | -0.43 |  | x | -0.26 |  | 0.88 |
| Fieldfare | count |  | 0.26 |  | 0.16 | 0.25 |  | 0.32 | 0.26 | -0.24 | 1.14 |
|  | zero |  | -1.20 |  | 0.49 | x |  | x | 0.40 | -0.17 | 1.26 |
| Ring ouzel | count |  | 0.38 |  | 0.28 | 0.96 | 0.66 | 0.43 |  |  | x |
|  | zero |  | -0.92 |  | 0.89 | x | 0.92 | x |  |  | 1.86 |
| Northern wheatear | count | 0.07 | -0.30 |  | -0.11 | 0.57 | 0.66 |  |  | x | 0.69 |
|  | zero | -0.44 | -0.57 |  | 1.27 | x | 0.74 |  |  | x | 0.44 |
| Willow warbler | count |  | 0.15 |  | 0.24 | 0.31 |  | 0.41 | 0.58 |  | 0.13 |
|  | zero |  | -1.58 |  | 0.45 | -0.55 |  | x | 0.31 |  | 1.09 |
| Northern raven | count |  | x |  | x | 0.06 | x | x |  |  | 0.05 |
|  | zero |  | -1.34 |  | 0.46 | x | 0.81 | 0.58 |  |  | x |
| Common redpoll | count |  | -0.10 |  | 0.10 | 0.15 |  | 0.18 |  |  | -0.42 |
|  | zero |  | -0.40 |  | 0.19 | -0.40 |  | x |  |  | -0.61 |
| Reed bunting | count | -0.11 | 0.78 | -0.07 | 0.59 | 0.15 | -0.41 |  |  | x | 0.41 |
|  | zero | -0.85 | -0.48 | -0.07 | 0.43 | -0.50 | 0.94 |  |  | -0.45 | 2.23 |
| Snow bunting | count |  | x |  | x | 0.54 | 1.01 |  |  |  | x |
| Lapland bunting | count |  | -0.30 |  | x | 0.23 | x |  |  |  | -1.79 |
|  | zero |  | -0.90 |  | 0.26 | -0.75 | 1.11 |  |  |  | x |

## Table S3: Regression coefficients for climate components

Regression coefficients for climate components for 39 species after variable selection. Covariates which were removed during variable selection are denoted by ‘x’. For each climate component (spatial, temporal, residual), temperature, precipitation and the interaction between temperature and precipitation were included. Species abundances were modelled using a negative bionimal distribution and a log link with or without zero-inflation with the exception of white-throated dipper for which a Poisson distribution was used. For species with zero-inflated models, the table contains separate rows for the count and the zero-inflation part of the model. For species with models without zero-inflation, the table contains one row for the counts only. Regression coefficients from models based on climate data from May-July of the current year are presented. For regression coefficients of land cover covariates, see Table S2.

|  |  | **Spatial** | | | **Temporal** | | | **Residual** | | |
| --- | --- | --- | --- | --- | --- | --- | --- | --- | --- | --- |
| **Species** | **Part** | **Temperature** | **Precipitation** | **Interaction** | **Temperature** | **Precipitation** | **Interaction** | **Temperature** | **Precipitation** | **Interaction** |
| Mallard | count | 0.76 | -0.33 | -0.33 | x | x | x | x | x | x |
|  | zero | -1.51 | x | x | 0.14 | x | x | 0.03 | 0.08 | 0.09 |
| Tufted duck | count | -1.31 | -0.67 | -0.46 | x | x | x | x | -0.11 | x |
|  | zero | 0.16 | 0.31 | 0.14 | 0.11 | 0.07 | -0.06 | 0.12 | x | x |
| Long-tailed duck | count | -2.42 | -0.90 | x | x | 0.14 | x | -0.12 | x | x |
| Red-breasted merganser | count | -0.54 | 0.15 | 0.29 | x | x | x | -0.02 | -0.08 | -0.07 |
|  | zero | -0.92 | 0.77 | 1.22 | 0.23 | 0.74 | -0.27 | x | x | x |
| Willow grouse | count | -1.56 | -0.08 | -0.25 | -0.02 | 0.01 | -0.05 | x | -0.07 | x |
| Rock ptarmigan | count | x | -0.10 | x | x | 0.11 | x | x | -0.12 | x |
|  | zero | 5.31 | 1.84 | 1.88 | -0.29 | x | x | x | x | x |
| Common ringed plover | count | -0.76 | -0.73 | x | 0.05 | -0.01 | 0.09 | x | x | x |
| Eurasian dotterel | count | -2.09 | -0.40 | x | 0.03 | 0.08 | 0.08 | 0.01 | -0.04 | -0.11 |
| Eurasian golden plover | count | -1.77 | 0.14 | 0.12 | 0.04 | 0.06 | x | x | x | x |
| Purple sandpiper | count | -1.77 | 0.41 | x | x | x | x | x | x | x |
| Ruff | count | -2.61 | -0.81 | x | 0.15 | 0.38 | x | x | x | x |
| Dunlin | count | -3.25 | -2.03 | -1.12 | 0.13 | 0.21 | x | x | 0.12 | x |
| Temminck's stint | count | -1.65 | x | x | x | x | x | x | x | x |
|  | zero | 0.91 | 1.91 | 0.88 | x | x | x | 0.15 | 0.52 | 0.43 |
| Wood sandpiper | count | -1.40 | 0.45 | x | 0.05 | 0.09 | 0.03 | x | -0.02 | x |
| Common redshank | count | -1.37 | -0.45 | -0.57 | 0.01 | -0.01 | 0.05 | -0.01 | -0.01 | -0.03 |
| Spotted redshank | count | -2.48 | -0.64 | 0.40 | x | x | x | x | x | x |
| Whimbrel | count | -1.97 | x | x | 0.09 | 0.06 | x | x | x | x |
| Common snipe | count | -0.21 | 0.07 | -0.21 | -0.11 | -0.09 | x | -0.05 | -0.01 | -0.02 |
|  | zero | -1.56 | x | x | x | x | x | x | x | x |
| Jack snipe | count | -2.58 | -1.34 | x | -0.15 | x | x | 0.12 | -0.14 | -0.20 |
| Red-necked phalarope | count | -3.13 | -1.30 | x | -0.30 | -0.15 | x | -0.29 | x | x |
| Long-tailed skua | count | -0.91 | -2.47 | -0.75 | 0.05 | 0.24 | -0.07 | 0.14 | 0.13 | -0.07 |
|  | zero | 6.23 | 0.71 | x | -1.11 | -1.00 | 0.74 | x | x | x |
| Common gull | count | 1.01 | -0.32 | -0.19 | -0.02 | 0.01 | -0.05 | x | x | x |
|  | zero | -1.72 | 0.97 | -0.12 | -0.19 | x | x | 0.15 | x | x |
| Arctic tern | count | -1.66 | -0.84 | -0.34 | x | -0.11 | x | x | x | x |
|  | zero | 0.50 | 1.06 | 0.70 | -0.16 | -0.30 | x | x | x | x |
| Common cuckoo | count | 0.58 | 0.03 | -0.23 | 0.02 | -0.01 | 0.01 | 0.03 | x | x |
|  | zero | -3.05 | 2.31 | 1.20 | x | x | x | 0.25 | 0.15 | -0.15 |
| Meadow pipit | count | -1.46 | -0.11 | -0.23 | x | x | x | x | x | x |
| Western yellow wagtail | count | -2.59 | -0.08 | -0.17 | 0.05 | 0.07 | x | x | -0.06 | x |
|  | zero | -4.97 | 2.12 | 1.01 | x | x | x | 0.27 | x | x |
| White-throated dipper | count | -0.99 | 0.28 | x | -0.11 | -0.14 | 0.07 | 0.00 | -0.08 | -0.06 |
| Bluethroat | count | -1.99 | -0.19 | x | -0.02 | 0.03 | 0.03 | x | x | x |
|  | zero | -1.12 | 2.53 | 0.91 | x | x | x | 0.14 | -0.17 | -0.15 |
| Whinchat | count | 0.28 | 0.40 | x | 0.04 | 0.03 | x | 0.02 | -0.01 | -0.02 |
|  | zero | -2.46 | 1.70 | 1.06 | -0.17 | 0.22 | 0.10 | x | x | x |
| Redwing | count | -0.30 | -0.25 | -0.18 | -0.04 | -0.05 | -0.04 | x | x | x |
|  | zero | -1.37 | 3.39 | 2.06 | x | x | x | 0.17 | 0.13 | x |
| Fieldfare | count | 0.31 | -0.39 | -0.22 | 0.04 | 0.00 | -0.02 | 0.02 | -0.02 | -0.03 |
|  | zero | -2.09 | 2.38 | 1.46 | 0.07 | 0.07 | 0.10 | 0.06 | 0.04 | -0.08 |
| Ring ouzel | count | 0.35 | -0.10 | -0.24 | 0.05 | 0.09 | x | 0.00 | -0.07 | -0.03 |
|  | zero | 2.50 | 0.39 | x | -0.07 | -0.31 | -0.19 | 0.19 | x | x |
| Northern wheatear | count | -0.83 | -0.36 | -0.26 | 0.04 | 0.08 | -0.02 | 0.03 | x | x |
|  | zero | -0.33 | 1.92 | 0.60 | -0.20 | x | x | 0.16 | 0.12 | -0.13 |
| Willow warbler | count | 0.41 | 0.05 | -0.06 | -0.02 | -0.01 | 0.01 | 0.00 | -0.01 | -0.01 |
|  | zero | -2.34 | 2.39 | 1.18 | x | x | x | 0.20 | 0.10 | x |
| Northern raven | count | 0.27 | -0.09 | x | -0.04 | -0.02 | 0.02 | x | x | x |
|  | zero | -0.14 | 2.13 | 0.76 | x | x | x | 0.41 | 0.28 | x |
| Common redpoll | count | -0.77 | 0.73 | 0.24 | -0.10 | -0.06 | x | 0.01 | -0.13 | -0.06 |
|  | zero | 0.92 | 0.20 | 0.48 | -0.06 | 0.11 | 0.07 | 0.09 | 0.02 | -0.17 |
| Reed bunting | count | -1.05 | -0.29 | -0.40 | -0.01 | -0.03 | -0.01 | 0.03 | 0.02 | -0.03 |
|  | zero | -3.11 | 3.82 | 1.78 | x | 0.33 | x | 0.13 | 0.08 | -0.11 |
| Snow bunting | count | -2.10 | x | x | -0.02 | 0.07 | -0.04 | x | 0.06 | x |
| Lapland bunting | count | -2.23 | -0.76 | x | 0.06 | x | x | 0.02 | 0.00 | -0.09 |
|  | zero | 4.53 | 3.11 | 1.50 | x | -0.25 | x | x | x | x |


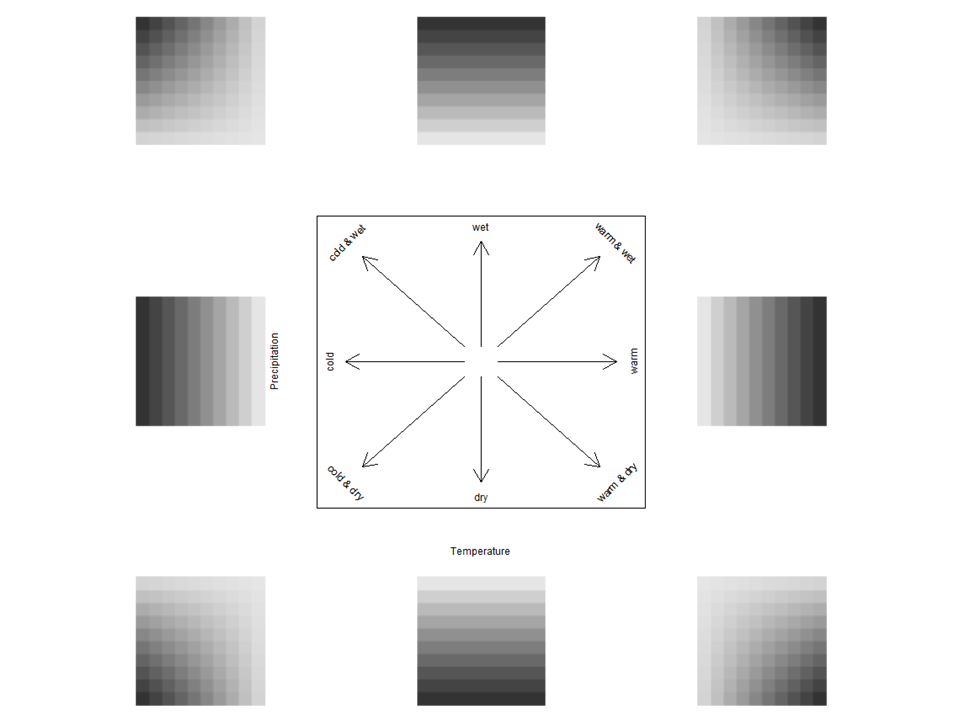


## Fig S7:

Conceptual graph for the predicted abundances of birds across the eight main domains (wet, warm-wet, warm, warm-dry, dry, cold-dry, cold, cold-wet) formed by temperature (x-axis) and precipitation (y-axis). Unimodal patterns are illustrated but bimodal or more complex patterns are also possible (Fig. S8).

| **Spatial** | **Temporal** | **Residual** | **Species** |
| --- | --- | --- | --- |
| **** | **** | **** | **Tufted duck** |
| **** | **** | **** | **Red-breasted merganser** |
| **** | **** | **** | **Common snipe** |
| **** | **** | **** | **Jack snipe** |
| **** | **** | **** | **Red-necked phalarope** |
| **** | **** | **** | **White-throated dipper** |
| **** | **** | **** | **Long-tailed duck** |
| **** | **** | **** | **Rock ptarmigan** |

Figure continued on next page.

| **Spatial** | **Temporal** | **Residual** | **Species** |
| --- | --- | --- | --- |
| **** | **** | **** | **Common ringed plover** |
| **** | **** | **** | **Eurasian dotterel** |
| **** | **** | **** | **European golden plover** |
| **** | **** | **** | **Ruff** |
| **** | **** | **** | **Dunlin** |
| **** | **** | **** | **Wood sandpiper** |
| **** | **** | **** | **Common redshank** |
| **** | **** | **** | **Whimbrel** |

Figure continued on next page.

| **Spatial** | **Temporal** | **Residual** | **Species** |
| --- | --- | --- | --- |
| **** | **** | **** | **Western yellow wagtail** |
| **** | **** | **** | **Bluethroat** |
| **** | **** | **** | **Whinchat** |
| **** | **** | **** | **Ring ouzel** |
| **** | **** | **** | **Northern wheatear** |
| **** | **** | **** | **Lapland bunting** |
| **** | **** | **** | **Purple sandpiper** |
| **** | **** | **** | **Temminck’s stint** |

Figure continued on next page.

| **Spatial** | **Temporal** | **Residual** | **Species** |
| --- | --- | --- | --- |
| **** | **** | **** | **Spotted redshank** |
| **** | **** | **** | **Meadow pipit** |
| **** | **** | **** | **Willow grouse** |
| **** | **** | **** | **Long-tailed skua** |
| **** | **** | **** | **Arctic tern** |
| **** | **** | **** | **Common cuckoo** |
| **** | **** | **** | **Redwing** |
| **** | **** | **** | **Reed bunting** |

Figure continued on next page.

| **Spatial** | **Temporal** | **Residual** | **Species** |
| --- | --- | --- | --- |
| **** | **** | **** | **Snow bunting** |
| **** |  |  | **Mallard** |
|  |  |  | **Common gull** |
|  |  |  | **Fieldfare** |
|  |  |  | **Willow warbler** |
|  |  |  | **Northern raven** |
|  |  |  | **Common redpoll** |

## Fig S8: Plots of predicted abundances of species for the spatial, temporal and residual climate component per species.

The plots correspond to the conceptual graph in Fig. S7 with temperature on the x-axis and precipitation on the y-axis. Predicted abundances are based on models with climate data from May-July of the current year.


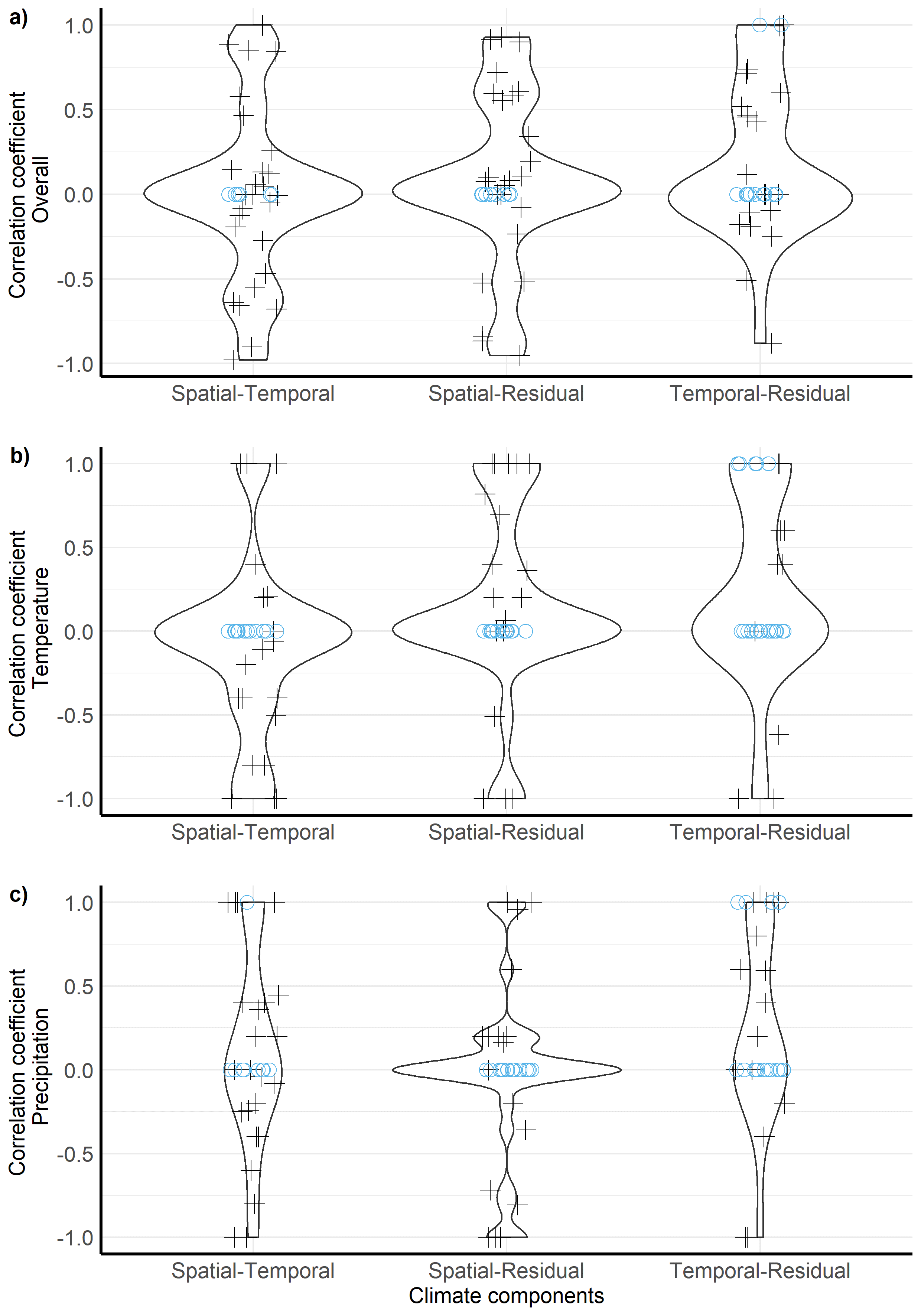


## Fig. S9: Correlations between the abundances predicted from any two climate components (spatial, temporal, and residual) indicate that the direction of effect was typically not consistent between any two climate components for models based on climate data from May-July of the previous year.

Violin plots of Spearman’s rank correlation coefficient between predicted local species abundances based on the spatial and temporal climate component, the spatial and residual climate components, and the temporal and residual components for a) both temperature and precipitation, b) temperature and c) precipitation. For a graphical description of how correlation coefficients were calculated, see Fig. S6. Violin plots show the probability density of the data at different values of the correlation coefficient. Black crosses and blue circles represent 33 individual species. Blue circles with correlation coefficients of zero represent species for which no association between a species local abundance and one of the climate components was found while an association was found with the other. Blue circles with correlation coefficients of one represent species for which no association between the local abundance of the species and both climate components were found. Thus, there is an agreement in conclusions of no effect of either climate component. Crosses represent species for which associations between the local abundance of the species and both climate components were found. High correlation coefficients indicate that predicted local abundances vary with the climate variable (temperature, precipitation or both) in the same direction for both climate components (spatial-temporal, spatial-residual or temporal-residual). Low correlation coefficients indicate that predicted local abundances vary with the climate variable in the opposite direction, for example local abundances may decrease with temperature for the spatial climate component but increase with the temporal climate component. Correlation coefficients at or near zero indicate that no association was found between the local abundance of the species and a climate component (blue circles) or that associations were complex, such as an increase of predicted local abundance with temperature at low precipitation, but a decrease at high precipitation (Fig. S6).

## S2: Results

### Decomposition models with climate data summarized from May-June of the current year

Model results were similar whether we summarized climate of the current year for May-June or for May-July. Regression coefficients for the nine climate components were highly correlated between both models (rank correlation coefficient for the coefficient estimates in full models: median: 0.92, min: 0.52, max: 1).

### Table S2

Retention of the spatial, temporal and residual climate components for the climate variables temperature and precipitation in models for 33 species with climate data summarized within May-June of the current year.

|  | Climate component | Number of species / % |
| --- | --- | --- |
| Temperature, | Spatial | 33 / 100 |
| precipitation | Temporal | 30 / 91 |
| or both | Residual | 25 / 76 |
| Temperature | Spatial | 33 / 100 |
|  | Temporal | 24 / 73 |
|  | Residual | 19 / 58 |
| Precipitation | Spatial | 32 / 97 |
|  | Temporal | 28 / 85 |
|  | Residual | 21 / 64 |
|  |  |  |


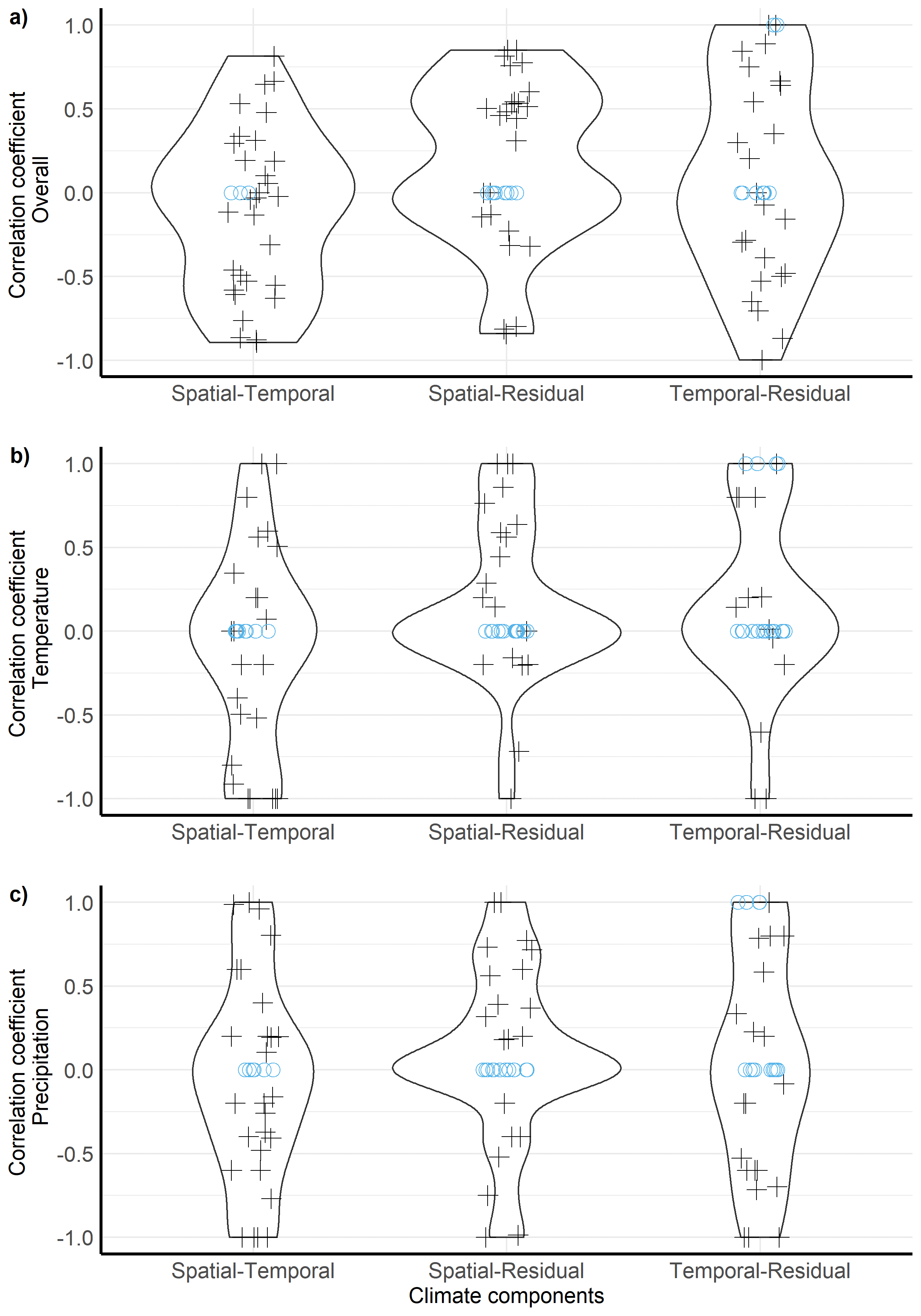


### Fig. S10: Correlations between the abundances predicted from any two climate components (spatial, temporal, and residual) indicate that the direction of effect was typically not consistent between any two climate components for models based on climate data from May-June of the current year.

Violin plots of Spearman’s rank correlation coefficient show rank correlation coefficients between the predicted local species abundances based on the spatial and temporal climate component, the spatial and residual climate components, and the temporal and residual components for a) both temperature and precipitation, b) temperature and c) precipitation. For a graphical description of how correlation coefficients were calculated, see Fig. S6. Violin plots show the probability density of the data at different values of the correlation coefficient. Black crosses and blue circles represent 33 individual species. Blue circles with correlation coefficients of zero represent species for which no association between a species local abundance and one of the climate components was found while an association was found with the other. Blue circles with correlation coefficients of one represent species for which no association between the local abundance of the species and both climate components were found. Thus, there is an agreement in conclusions of no effect of either climate component. Crosses represent species for which associations between the local abundance of the species and both climate components were found. High correlation coefficients indicate that predicted local abundances vary with the climate variable (temperature, precipitation, or both) in the same direction for both climate components (spatial-temporal, spatial-residual, or temporal-residual). Low correlation coefficients indicate that predicted local abundances vary with the climate variable in the opposite direction, for example local abundances may decrease with temperature for the spatial climate component but increase with the temporal climate component. Correlation coefficients at or near zero indicate that no association was found between the local abundance of the species and a climate component (blue circles) or that associations were complex, such as an increase of predicted local abundance with temperature at low precipitation, but a decrease at high precipitation (Fig. S6).
